# G3’M_TMD3_ in the insect GABA receptor subunit, RDL, confers resistance to broflanilide and fluralaner

**DOI:** 10.1371/journal.pgen.1010814

**Published:** 2023-06-29

**Authors:** Yichi Zhang, Qiutang Huang, Chengwang Sheng, Genyan Liu, Kexin Zhang, Zhongqiang Jia, Tao Tang, Xin Mao, Andrew K. Jones, Zhaojun Han, Chunqing Zhao

**Affiliations:** 1 Key Laboratory of Integrated Pest Management on Crops in East China, Ministry of Agriculture, Nanjing Agricultural University, Nanjing, People’s Republic of China; 2 Key Laboratory for Green Chemical Process of Ministry of Education, School of Chemical Engineering and Pharmacy, Wuhan Institute of Technology, Wuhan, People’s Republic of China; 3 Institute of Plant Protection, Hunan Academy of Agricultural Sciences, Changsha, People’s Republic of China; 4 Department of Biological and Medical Sciences, Faculty of Health and Life Sciences, Oxford Brookes University, Oxford, United Kingdom; University of Kentucky, UNITED STATES

## Abstract

Meta-diamides (e.g. broflanilide) and isoxazolines (e.g. fluralaner) are novel insecticides that target the resistant to dieldrin (RDL) subunit of insect γ-aminobutyric acid receptors (GABARs). In this study, we used *in silico* analysis to identify residues that are critical for the interaction between RDL and these insecticides. Substitution of glycine at the third position (G3’) in the third transmembrane domain (TMD3) with methionine (G3’M _TMD3_), which is present in vertebrate GABARs, had the strongest effect on fluralaner binding. This was confirmed by expression of RDL from the rice stem borer, *Chilo suppressalis* (*Cs*RDL) in oocytes of the African clawed frog, *Xenopus laevis*, where the G3’M_TMD3_ mutation almost abolished the antagonistic action of fluralaner. Subsequently, G3’M_TMD3_ was introduced into the *Rdl* gene of the fruit fly, *Drosophila melanogaster*, using the CRISPR/Cas9 system. Larvae of heterozygous lines bearing G3’M_TMD3_ did not show significant resistance to avermectin, fipronil, broflanilide, and fluralaner. However, larvae homozygous for G3’M_TMD3_ were highly resistant to broflanilide and fluralaner whilst still being sensitive to fipronil and avermectin. Also, homozygous lines showed severely impaired locomotivity and did not survive to the pupal stage, indicating a significant fitness cost associated with G3’M_TMD3_. Moreover, the M3’G_TMD3_ mutation in the mouse *Mus musculus* α1β2 GABAR increased sensitivity to fluralaner. Taken together, these results provide convincing *in vitro* and *in vivo* evidence for both broflanilide and fluralaner acting on the same amino acid site, as well as insights into potential mechanisms leading to target-site resistance to these insecticides. In addition, our findings could guide further modification of isoxazolines to achieve higher selectivity for the control of insect pests with minimal effects on mammals.

## Author summary

Meta-diamides (e.g. broflanilide) and isoxazolines (e.g. fluralaner) are members of group 30 of compounds according to the Insecticide Resistance Action Committee, and are defined as γ-aminobutyric acid (GABA)-gated chloride channel allosteric modulators. However, their mode of action on the GABAR remains to be fully elucidated. In the current study, we provide *in silico*, *in vitro* and *in vivo* evidence that G3’_TMD3_, the glycine residue at the third position (G3’) in the third transmembrane domain (TMD3) of the insect GABAR subunit, resistant to dieldrin (RDL), is critical for the action of these insecticides. Furthermore, the homozygous but not heterozygous lines of the fruit fly *Drosophila melanogaster* bearing G3’M_TMD3_ showed high resistance to broflanilide and fluralaner but were unable to survive to the pupal stage. These findings enhance our understanding of resistance-related and homozygous-lethal gene mutations, and may prove useful for the future synthesis of related insecticides to gain long-term effectiveness and higher selectivity.

## Introduction

High and efficient agricultural activity is required to meet the demand of an ever-growing human population. However, agricultural productivity is hampered by insect pests, which can lead to 20%-30% loss of crops [1]. To date, insecticides are still the most widely used tool for controlling insect pests. However, crop protection efforts are undermined by the development of resistance to insecticides. Exploring the molecular targets of insecticides forms an important basis for understanding mechanisms leading to resistance [2].

The insect γ-aminobutyric acid receptor (GABAR) subunit, RDL (resistant to dieldrin), is the molecular target for various types of insecticides, such as cyclodienes, phenylpyrazoles, macrocyclic lactones, meta-diamides and isoxazolines [3] (**S1 Fig**). Meta-diamides and isoxazolines are novel classes of compounds, which have been classified into group 30 by the Insecticide Resistance Action Committee, and are used to protect crops and animals from insect pests [4]. Despite acting on the same molecular target, meta-diamides and isoxazolines do not show cross-resistance with cyclodienes or phenylpyrazoles [3]. Previous studies demonstrated that mutations at A302 (the fruit fly, *Drosophila melanogaster*, numbering, otherwise referred to as A2’_TMD2_) in the second transmembrane domain (TMD2) of RDL underlie resistance to fipronil and dieldrin [3,5,6]. In contrast, the potency of meta-diamides and isoxazolines is unaffected by mutations at A2’_TMD2_, suggesting that they act on a site different to that of cyclodienes or phenylpyrazoles [7–10]. In line with this, G3’M_TMD3_, a mutation in TMD3 of heterologously expressed RDL, substantially reduced the potency of meta-diamides and isoxazolines (**S2 Fig**) [10–13]. Attempts to generate diamondback moth *Plutella xylostella* that were resistant to broflanilide were unsuccessful after ten generations of selection [14]. Thus, potential mechanisms underlying resistance to meta-diamides, including mutations in RDL, remain to be identified *in vivo*.

In the present study, differences between arthropod RDL and mammalian GABAR subunits (e.g. α, β and γ) were explored to highlight and reinforce residues in the TMDs as being important for the interaction between GABARs and meta-diamides (e.g. broflanilide) or isoxazolines (e.g. fluralaner). Also, *D*. *melanogaster*, the classical insect model organism, was edited using the CRISPR/Cas9 system to determine whether mutations at G3’_TMD3_ in RDL *in vivo* lead to resistance to broflanilide and fluralaner. Knowledge gained from this study can guide further modification of novel insecticides to achieve highly selective toxicity to insect pests with minimal effect on mammals, as well as provide insights into a potential mechanism underlying resistance to meta-diamides and isoxazolines in the field.

## Results

### Prediction of potential binding sites of fluralaner

Amino acid residues of arthropod RDL and vertebrate GABAR subunits were aligned and the four TMDs were identified (**S3 and S4 Figs and S1 Table and S1 Note**). The effect of mutant residues in the TMDs on binding of fluralaner to a three-dimensional homology model of RDL from the rice stem borer, *Chilo suppressalis*, was assessed. Altering several residues in RDL to the equivalent amino acid present in vertebrate GABARs increased the binding energy of fluralaner (**S1 Table**). Twelve of these mutations were selected for functional expression in oocytes of the African clawed frog, *Xenopus laevis*, in order to determine if they play a role in the interaction between RDL and fluralaner (**S1 Table**).

### Electrophysiological responses of wild-type and mutant *Cs*RDL to GABA and fluralaner

**Fig 1:**
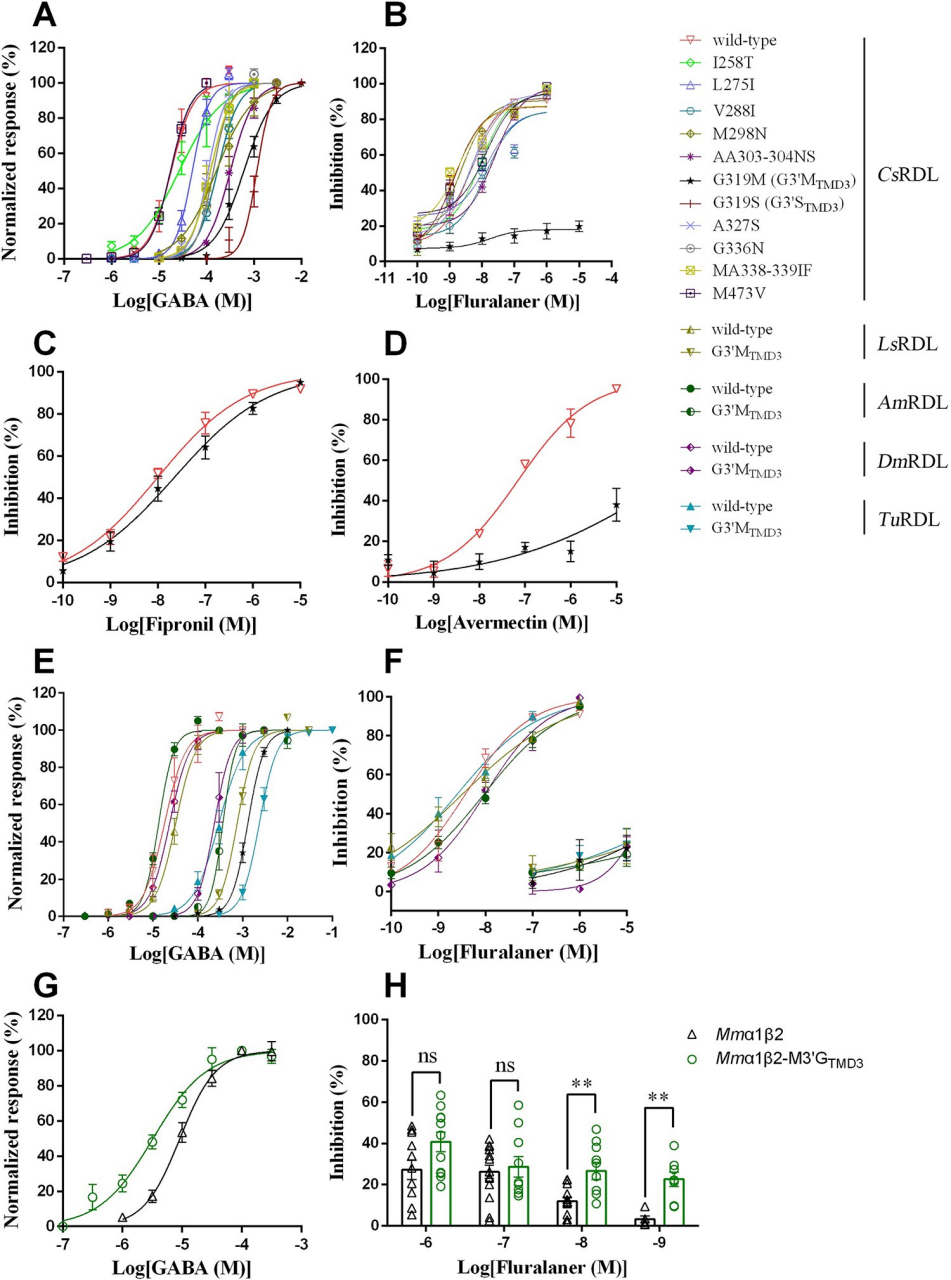
Responses to GABA and antagonists in *Xenopus laevis* oocytes expressing wild-type and mutant arthropod or mammalian GABAR subunits. (**A**) and (**B**) Concentration-response curves of GABA (A) and inhibition of GABA-induced currents by fluralaner (B) from wild-type or mutant RDL receptors. (**C**) and (**D**) Inhibition of GABA-induced currents by fipronil (C) and avermectin (D) in wild-type or G3’M_TMD3_ RDL. (**E**) and (**F**) Effect of the G3’M_TMD3_ mutation in RDL of different arthropod species on response to GABA (E) or fluralaner (F). (**G**) and (**H**) Concentration-response curves of GABA (G) and inhibition of GABA-induced currents by fluralaner (H) from heteromeric *Mm*α1β2 or mutant *Mm*α1β2-M3’G_TMD3_ channels. Error bars indicated the standard error of the mean (SE). Significant difference was determined by Student’s *t*-test (ns, not significant; **, *P* < 0.01).

Twelve homomeric mutant *C*. *suppressalis* (*Cs*RDL) subunits were expressed in *X*. *laevis* oocytes (**S2 Table**). Inward currents upon GABA application were not detected for one mutant, I477D. The maximum GABA-induced current (*I*_max_) was notably lower in the mutant channels than that of the wild-type *Cs*RDL channel except for G3’M_TMD3_. All mutations except for I258T and M473V decreased the potency of GABA as indicated by a significant increase in EC_50_ (**Figs 1A and S5 and S2 Table**). The EC_50_ values of G3’M_TMD3_ and G3’S_TMD3_ increased 34- and 64-fold, respectively, compared with that of the wild-type *Cs*RDL channel (**Fig 1A and S2 Table**).

Fluralaner showed concentration-dependent antagonistic action on the GABA (EC_50_)-induced currents (**Figs 1B and S5**) with an IC_50_ of 4.20 nM in the wild-type *Cs*RDL channel (**S3 Table**). The double-mutation (AA303-304NS) caused a significant 5.91-fold increase in IC_50_, whilst G3’M_TMD3_ decreased the potency of fluralaner even further with an IC_50_ > 10,000 nM.

### G3’M_TMD3_ reduces the sensitivity of RDL to avermectin

Both fipronil and avermectin strongly inhibited the GABA-induced current in the homomeric wild-type *Cs*RDL channel with an IC_50_ of 10.02 and 69.90 nM, respectively (**Fig 1 and S4 Table**). However, the G3’M_TMD3_ *Cs*RDL channel was less sensitive than the wild-type to fipronil or avermectin as indicated by significantly greater IC_50_ values. In particular, avermectin at 10^−5^ M only inhibited 38.06% of the GABA-induced response in *Cs*RDL with the G3’M_TMD3_ as opposed to almost abolishing the GABA response in wild-type channels (**Fig 1D**).

### G3’M_TMD3_ reduces the sensitivity of arthropod RDL to fluralaner

To verify whether G3’M_TMD3_ can potentially give rise to resistance to fluralaner in different arthropod species, the mutation was introduced into RDL from Hymenoptera (*Apis mellifera*), Hemiptera (*Laodelphax striatellus*), Arachnoidea (*Tetranychus urticae*), Lepidoptera (*C*. *suppressalis*), and Diptera (*D*. *melanogaster*). The mutant RDL subunits were individually expressed in *X*. *laevis* oocytes. In each case, G3’M_TMD3_ reduced the potency of GABA by 8–34-fold and showed significantly different EC_50_ values compared with that of the corresponding wild-type RDL (**Fig 1E and S5 Table**). In addition, the G3’M_TMD3_ in RDL of each species reduced the potency of fluralaner as shown by significantly increased IC_50_ values (**Fig 1F and S5 Table**).

### M3’G_TMD3_ in the *Mm*α1β2 GABAR affects the potency of fluralaner

To examine the contribution of G3’_TMD3_ of mammalian GABARs towards fluralaner insensitivity, the M3’G_TMD3_ was introduced into the mouse *Mus musculus* GABAR β2 subunit (*Mm*β2). There was no detectable response to GABA in *X*. *laevis* oocytes injected with *Mm*α1 or *Mm*β2 alone. However, co-expression of *Mm*α1 and *Mm*β2 subunits generated a functional heteromeric GABA-gated channel (*Mm*α1β2) (**Fig 1 and S6 Table**). GABA potency on the *Mm*α1β2-M3’G_TMD3_ channel was enhanced as shown by a significantly lower EC_50_ value compared to that of the wild-type (**Fig 1G and S6 Table**). Fluralaner showed decreased potency on *Mm*β2-M3’G_TMD3_ at concentrations of 10^−8^ and 10^−9^ M as shown by significantly less inhibition of GABA-induced currents (**Fig 1H and S7 Table**).

### Generation of genome-modified *D*. *melanogaster* bearing mutations at G3’_TMD3_

**Fig 2:**
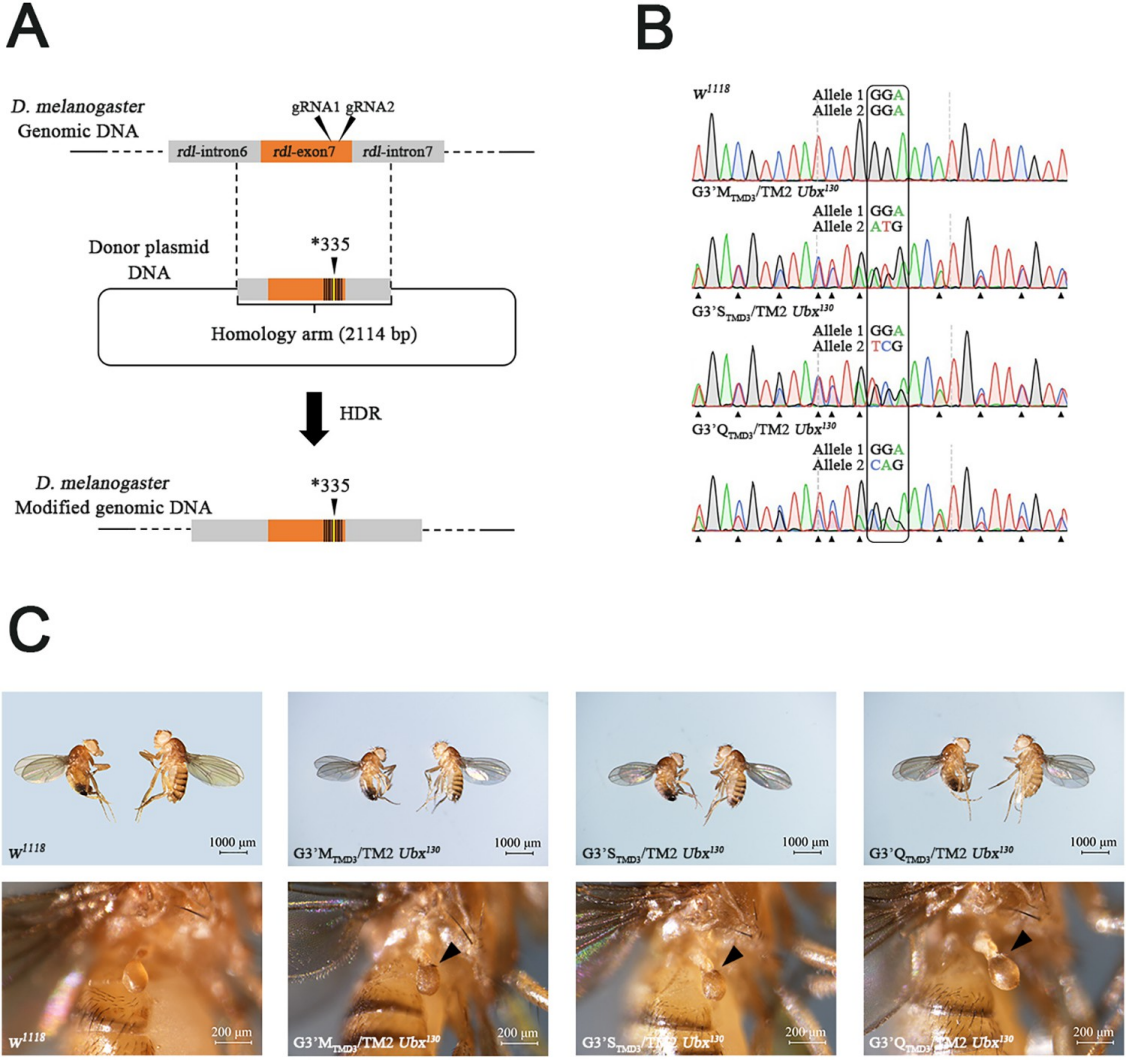
CRISPR/Cas9-mediated point-mutation at G3’_TMD3_ in the *Rdl* gene of *Drosophila melanogaster*. (A) Diagram of the genome editing strategy. Black triangles indicated the gRNA-targeted sites. A 2114 bp homologous region with a modified codon corresponding to the G3’_TMD3_ residue was cloned into the donor plasmid, and nine synonymous mutations indicated by vertical lines were designed around the G3’_TMD3_ residue to prevent repeated editing. (B) Genotypes of the heterozygous mutant strains were confirmed by sequencing of genomic DNA. The corresponding codon of the G3’_TMD3_ residue is boxed, and the synonymous mutations are indicated by black equilateral triangles. (C) Balancer-associated phenotype of heterozygous mutant strains. Additional bristles on the haltere are indicated by a black triangle.

The CRISPR/Cas9 genome editing system was used to introduce substitutions at G3’_TMD3_ (G335) in *Dm*RDL (**Figs 2A and S6**). For the G3’M_TMD3_ or G3’S_TMD3_, a mixture including Cas9 mRNA, donor plasmid and gRNAs was injected into the embryos of the *w*^*1118*^ strain (defined as G_0_ generation). In the G_0_ generation, 2 out of 12 or 4 out of 25 individuals carrying the G3’M_TMD3_ or G3’S_TMD3_ allele, respectively, were identified. Subsequently, the G_1_ individuals from these positive lines were crossed with the balancer strain (*w*^*1118*^; TM2 *Ubx*^*130*^/TM6B *Tb*^*1*^) to retain the mutant allele in the G_2_ generation (**S7 Fig**). After self-crossing of G_2_ generation, G3’M_TMD3_ or G3’S_TMD3_ homozygous lines were not observed in the progeny of the G_3_ generation, indicating that either mutation at G3’_TMD3_ may cause homozygous-lethality in *D*. *melanogaster* adults. Therefore, strains carrying the TM2 *Ubx*^*130*^ balancer chromosome, which are also heterozygous for G3’M_TMD3_ or G3’S_TMD3_, were generated and verified by genomic DNA sequencing (**Fig 2**).

Because G3’Q_TMD3_ homozygous lines could not be generated with the *w*^*1118*^ strain, the nos.Cas9 strain with a different genetic background in the X chromosome was used for establishing the G3’Q_TMD3_ homozygous lines injected with specific gRNAs/donor plasmid mixture (**S6 Fig**), which was defined as G_0nos_. The G_0nos_ adults were individually crossed with the nos.Cas9 flies, and the G_1nos_ progeny were identified by genomic DNA sequencing. The G3’Q_TMD3_ mutation was detected in 1 out of 18 G_1nos_ lines, indicating that the homology-directed repaired (HDR) allele (G3’Q_TMD3_) was present in the G_0nos_ strain. Unfortunately, similar to G3’M_TMD3_ and G3’S_TMD3_, G3’Q_TMD3_ was homozygous lethal. Therefore, a heterozygous G3’Q_TMD3_ strain was used for further study (**Fig 2**).

In the complementation experiment, all progeny produced by these crosses carried the parental balancer chromosomes, thus no complementation was apparently viable. This result confirmed that the observed lethality was linked to the corresponding genomic region containing *Rdl*, presumably to G3’M/S/Q_TMD3_. Therefore, we concluded that the substitutions at G3’_TMD3_ of the RDL subunit might have a severe impact on channel function affecting viability.

### Heterozygous adults bearing a mutation at G3’_TMD3_ were not resistant to insecticides targeting the GABAR

Heterozygous *D*. *melanogaster* adults bearing a mutation at G3’_TMD3_ (G3’M_TMD3_, G3’Q_TMD3_ or G3’S_TMD3_) were sensitive to fipronil, fluralaner, broflanilide and avermectin without significant alteration in resistance ratio (**S8 Table**). The findings in the current and previous studies indicated that mutations at G3’_TMD3_ reduced potency of these insecticides when using *in vitro* and *in silico* approaches [10,12,15].

### Temporal characteristics of lethality caused by homozygous mutations at G3’_TMD3_

**Fig 3:**
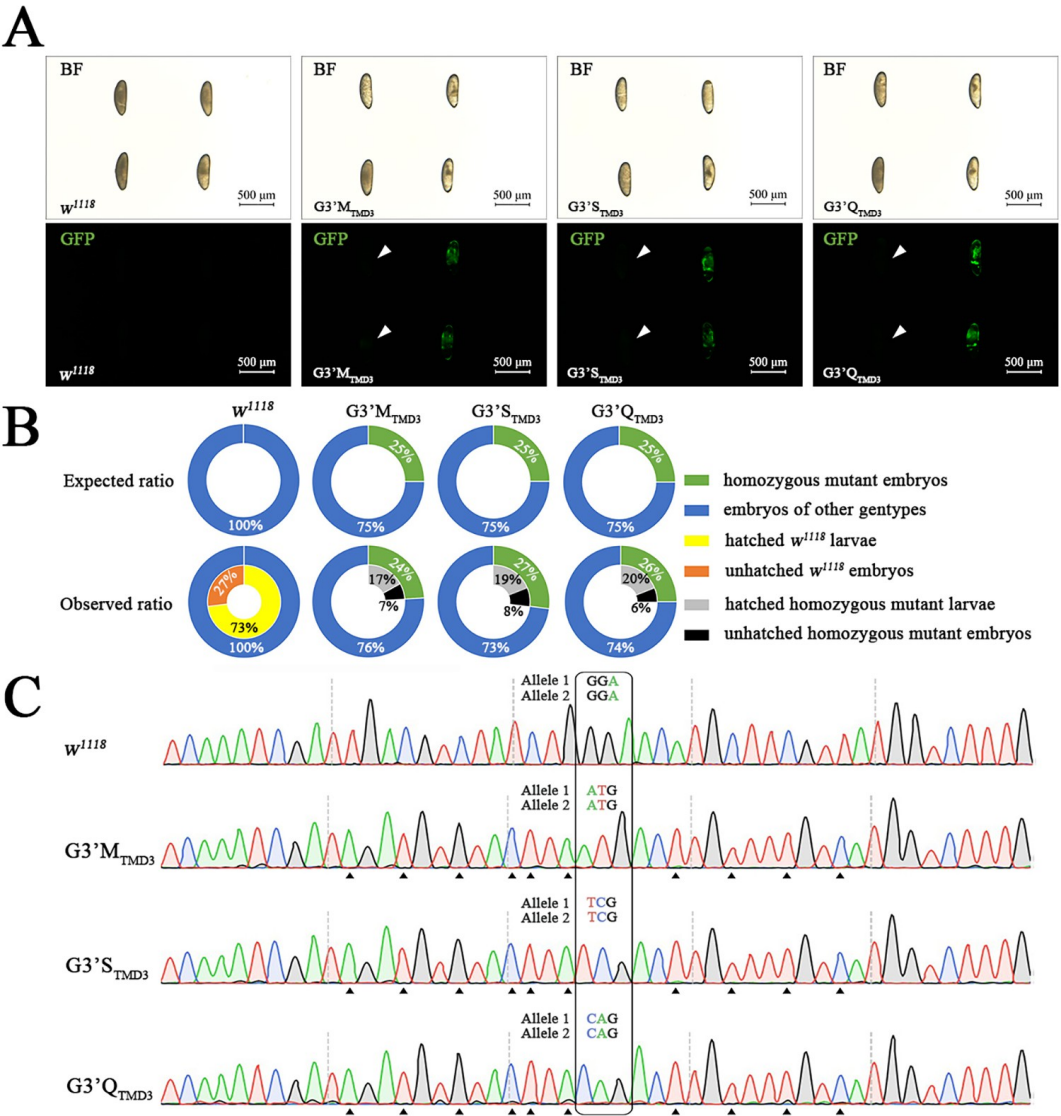
Identification of *D*. *melanogaster* embryos bearing homozygous mutations at RDL G3’_TMD3_. (A) Fluorescence detection of embryos. Heterozygous mutant or TM3 *Sb GFP*/ TM3 *Sb GFP* embryos showing a GFP signal. Homozygous mutant embryos labeled with white triangles showed no GFP signal. BF, bright field. (B) The proportion and hatching rate of embryos bearing homozygous mutations. For each strain, genotypes of 100 embryos were identified. 70.83% (17 of 24), 70.37% (19 of 27) and 76.92% (20 of 26) embryos hatched that were homozygous for G3’M_TMD3_, G3’S_TMD3_ and G3’Q_TMD3_, respectively. (C) Genotypes of the homozygous mutant larvae were confirmed by sequencing of genomic DNA. The codon of the G3’_TMD3_ residue is boxed and the synonymous mutations are labeled with black equilateral triangles.

In order to further investigate the substitution-associated lethality of homozygous mutations at G3’_TMD3_ in RDL, we crossed the heterozygous mutants (G3’M_TMD3_/TM2 *Ubx*^*130*^, G3’S_TMD3_/TM2 *Ubx*^*130*^ or G3’Q_TMD3_/TM2 *Ubx*^*130*^) with the *w*^*1118*^; dIRE1^Δ^/TM3 *Sb GFP* strain to obtain mutants carrying a GFP-labeled balancer. Analysis of embryos showed that the GFP and homozygous mutations at G3’_TMD3_ in RDL did not hinder embryogenesis of *D*. *melanogaster* (**Fig 3A**). The proportion of embryos homozygous for G3’M_TMD3_, G3’S_TMD3_ or G3’Q_TMD3_ was 24%, 27% and 26%, respectively (**Fig 3B**). Furthermore, their corresponding hatching rate was 70.83%, 70.37% and 76.92%, respectively, and that of *w*^*1118*^ was 73.00%. These results demonstrated that the homozygous embryos of G3’M_TMD3_, G3’S_TMD3_ or G3’Q_TMD3_ could develop and hatch normally.

**Fig 4:**
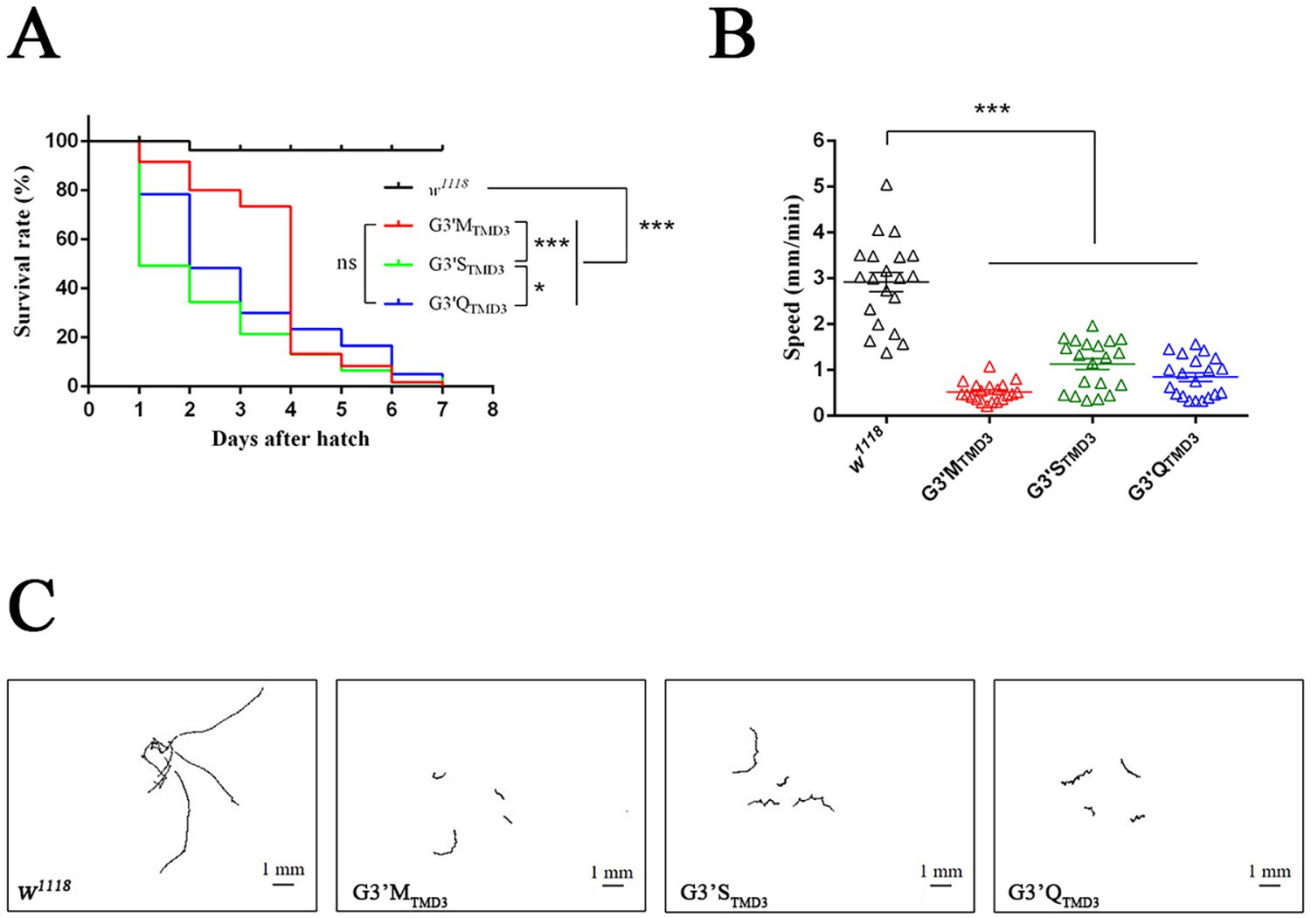
Lethality and reduced locomotion in *D*. *melanogaster* larvae caused by homozygous mutations at G3’_TMD3_. (A) Temporal characteristics of lethality in homozygous mutants. Data were analyzed using the Log-rank test for trend and the Mantel-Cox test (ns, not significant; *, *P* < 0.05; ***, *P* < 0.001). (B) Crawling speed of homozygous mutants. Error bars indicate the standard error of the mean (SE). Significant difference was determined by Student’s *t*-test (***, *P* < 0.001). (C) Representative motion path of homozygous mutants in 2 minutes (**S1 Video**).

After hatching of the three homozygous G3’M_TMD3_ strains of *D*. *melanogaster*, a significant difference was observed in their survival rate (**Fig 4A and S9 Table**). Approximate 90% of their larvae survived for one day and > 70% for three days. However, only 30% and 20% of homozygous G3’Q_TMD3_ and G3’S_TMD3_ larvae, respectively, survived for three days. All homozygous mutant flies stayed at the larval stage until death within 7 days, whilst the control strain, *w*^*1118*^, progressed to pupation.

### Larvae bearing the homozygous G3’M_TMD3_ were resistant to fluralaner and broflanilide

Because the survival rate of the homozygous G3’M_TMD3_ larvae was higher than 90% at 24 h after hatching (**Fig 4A**), the sensitivity of newly-hatched larvae towards fluralaner and broflanilide were measured. As shown in **Table 1**, LC_50_ values of fluralaner and broflanilide for the *w*^*1118*^ larvae were 1.08 and 0.47 mg/kg, respectively. In contrast, the mortality of the homozygous G3’M_TMD3_ larvae was 35.71% and 39.54% to fluralaner and broflanilide at 1000 mg/kg, respectively, which indicated that they have developed > 900-fold resistance to these two insecticides compared to the *w*^*1118*^ larvae. Such high levels of cross-resistance suggested that the G3’_TMD3_ residue plays a critical function in the interaction between RDL and fluralaner or broflanilide *in vivo*. In addition, it is worth noting that the homozygous G3’M_TMD3_ larvae also showed resistance (6.80-fold) to avermectin but not to fipronil.

**Table 1:** Sensitivity of the wild-type *w*^*1118*^ larvae and homozygous G3’M_TMD3_ larvae to four different insecticides.

Insecticide	Genotype	Slope ± SE	LC_50_ (95% CI ^†^) (mg/kg)	χ^2^ (df)	*P* value	RR^‡^
Fipronil	*w* ^*1118*^	2.03±0.27	0.31 (0.22–0.40)	2.51 (13)	0.999	1.00
	G3’M_TMD3_	1.49±0.20	0.31 (0.20–0.44)	4.77 (13)	0.980	1.00
Avermectin	*w* ^*1118*^	2.67±0.38	0.10 (0.08–0.11)	8.59 (13)	0.803	1.00
	G3’M_TMD3_	1.63±0.24	0.68*(0.49–0.89)	3.98 (13)	0.991	6.80
Fluralaner	*w* ^*1118*^	1.14±0.14	1.08 (0.73–1.57)	5.02 (13)	0.975	1.00
	G3’M_TMD3_	—	>1000*	—	—	> 925.93
Broflanilide	*w* ^*1118*^	0.87±0.13	0.47 (0.25–0.76)	4.66 (13)	0.982	1.00
	G3’M_TMD3_	—	> 1000*	—	—	> 2127.66

^†^ 95% confidence interval.

* indicates significant difference relative to the wild-type *w*^*1118*^ as determined by the 95% CI without overlapping.

^‡^ RR indicates resistance ratio: resistance ratio = LC_50_ value of the G3’M_TMD3_/LC_50_ value of the wild-type *w*^*1118*^.

### Larvae homozygous for G3’M_TMD3_ displayed less locomotion

A crawling assay was used to investigate the potential fitness cost caused by homozygous mutations at the RDL G3’_TMD3_ residue. As shown in **Fig 4 and S1 Video**, *D*. *melanogaster* larvae homozygous for G3’M_TMD3_, G3’Q_TMD3_ or G3’S_TMD3_ showed an average speed of 0.52, 0.85 and 1.13 mm/min, respectively (**S10 Table**). In contrast, the wild-type *w*^*1118*^ larvae were faster at 2.92 mm/min (**Fig 4B and 4C**), indicating a significant mutation-associated decrease in locomotion.

## Discussion

To date, insects have developed varying levels of resistance to traditional GABAR-targeting insecticides such as cyclodienes, phenylpyrazoles and macrocyclic lactones [5,16–18]. Previous studies investigating mechanisms of resistance have provided insights into their binding site(s). For example, A2’_TMD2_ of RDL is recognized as the common binding site of cyclodienes and phenylpyrazoles. A mutation at A2’_TMD2_ is the critical basis for resistance to dieldrin (A2’S_TMD2_) or fipronil (A2’N_TMD2_) [19–23]. However, RDL with resistance-related mutations of A2’S/G/N_TMD2_ is still inhibited by fluralaner [24–28].

Meta-diamides (e.g. broflanilide) and isoxazolines (e.g. fluralaner) are defined as non-competitive antagonist (NCA)-II compounds that share a coupled action site in proximity to the interface of TMD1/TMD3 in RDL [3,29]. In the current study, the amino acid sequences of arthropod and mammalian GABARs were aligned in order to explore the potential binding site of fluralaner. A similar research approach was performed on the bumblebee *Bombus impatiens Bi*Na_v_1 sodium channel, which identified additional amino acid residues that underlie the sensitivity of *B*. *impatiens* to pyrethroids as well as selective resistance to tau-fluvalinate [30]. With mammals being relatively insensitive to fluralaner [24,31], we identified 35 amino acid residues in the TMDs that differ between arthropod and mammalian GABAR subunits (**S1 Table**). Heterologous expression studies showed that the G3’M_TMD3_ mutation led to the greatest decrease in fluralaner potency (**S3 Table**) reinforcing this residue as being crucial for isoxazoline activity, which is consistent with previous studies [10,12]. Meanwhile, G3’M_TMD3_ also considerably decreased the antagonist actions of avermectin (**S4 Table**). Avermectin is a macrocyclic lactone and is thought to act primarily on the glutamate-gated chloride channel (GluCl) with GABARs being a secondary target [32]. In line with this, both G323D in *Tu*GluCl and G315E in *Px*GluCl (equivalent position to RDL G3’_TMD3_) are associated with resistance to avermectin [33,34]. Although the G3’M mutation might allosterically affect GABA binding to the orthosteric site leading to the significantly different EC_50_ values compared to that of wild-type (**S2 Table**), the concentration-response curve indicated that the channel was still able to function in response to GABA (**Fig 1A and 1B**). Other studies found that fluralaner [11,25] and avermectin inhibited [^3^H]fluralaner binding on *M*. *domestica* membranes [28]. Fluralaner binding was also inhibited when the G3’M_TMD3_ was present in the homomeric RDL channels of the common cutworm, *Spodoptera litura*, and *D*. *melanogaster* [10,11].

Desmethyl-broflanilide, which has a common genesis with broflanilide, has a site of action near G3’_TMD3_ in the *Dm*RDL subunit [10,35]. Nakao et al. (2013) reported that the volume of amino acid at G3’_TMD3_ is a factor that determines the inhibitory activity of desmethyl-broflanilide [10]. Therefore, three mutations (G3’M/S/Q_TMD3_), which eliminate the insecticidal sensitivity with only a minor change in activity of GABAR, were selected for further assay *in vivo* in this study.

CRISPR/Cas9 is a powerful tool for generating specific mutations to validate gene function, e.g. insecticide resistance in *D*. *melanogaster* [36–41]. So far, several studies adopting this “reverse genetic” approach have successfully demonstrated the mode of action of various types of insecticides by modifying target receptors with compelling association between mutations and phenotypes [42–46]. The mutation of G3’M_TMD3_ or G3’S_TMD3_ was successfully introduced into *w*^*1118*^ *Dm*RDL and heterozygote lines were established. Meanwhile, the G3’Q_TMD3_ was successfully introduced into the nos.Cas9 strain. Finally, nos.Cas9 bearing G3’Q_TMD3_ in the X chromosome was replaced with the *w*^*1118*^ allele during crossing ensuring that the three mutant (G3’M/S/orQ_TMD3_) strains were generated with a consistent genetic background. Unfortunately, introduction of G3’M/S/orQ_TMD3_ resulted in lethality making it impossible to obtain the homozygous mutant strains. This may be a reason for the failure of selecting broflanilide-resistant strains of *P*. *xylostella* [14]. Similarly, occurrence of homozygous-lethal effects have hampered investigations into the modes of action of particular insecticides using the CRISPR/Cas9 strategy [45,47,48]. Therefore, the G3’M_TMD3_, G3’S_TMD3_ and G3’Q_TMD3_ were maintained in heterozygous strains, and the direct linkage between homozygous lethality and target-site mutations was proven by a complementation experiment [49].

The GFP-labeled allele can be used as a useful tool to identify the homozygote and heterozygote mutants at the embryo stage of *D*. *melanogaster*, especially when the phenotypes of the gene mutations are unknown [50,51]. Thus, heterozygous mutant strains with a GFP-labeled balancer allele enabled accurate selection of homozygous G3’_TMD3_ mutants by fluorescence detection. Embryos bearing any of the three homozygous mutations at RDL G3’_TMD3_ were able to hatch where 25% of embryos from each heterozygous strain were expected to carry homozygous mutations [52]. In line with this, the proportion of homozygous G3’M_TMD3_, G3’S_TMD3_ and G3’Q_TMD3_ embryos was 24%-27% (**Fig 3B**). It is worth noting that the temporal characteristics of lethality varied among the three mutants during the first three days after hatching (**Fig 4A**).

In this study, heterozygous lines bearing mutations at G3’_TMD3_ did not show significant resistance to broflanilide or fluralaner (**S8 Table**). This is in line with a previous study using electrophysiology, where expression of heterozygous G3’_TMD3_ mutant RDL in *D*. *melanogaster* Mel-2 cells did not confer resistance to demethyl-broflanilide compared with the wild-type RDL alone [15]. Therefore, these *in vivo* and *in vitro* studies indicate that heterozygous mutations at RDL G3’_TMD3_ do not confer resistance to meta-diamides and isoxazolines. *Drosophila melanogaster* homozygous for G3’M_TMD3_ were selected at the larval stage for bioassays due to their low mortality during the first three days after hatching. These larvae were highly resistant to broflanilide and fluralaner with LC_50_ > 1000 mg/kg, providing convincing evidence *in vivo* that meta-diamides and isoxazolines share the same mode of action and directly interact with the RDL subunit. It is also worth noting that larvae homozygous for G3’M_TMD3_ showed low-level resistance to avermectin, indicating that avermectin might share an overlapping but weak binding mode with fluralaner and broflanilide on the GABAR. However, the toxicity of fipronil was not affected by the homozygous G3’M_TMD3_ mutation, which indicated a different binding site, in accord with previous studies *in vivo* and *in vitro* [6,11,22,23].

It has been previously reported that the knock-down of RDL can affect the locomotivity of *D*. *melanogaster* [53]. In this study, our results showed that *D*. *melanogaster* larvae homozygous for G3’M/S/orQ_TMD3_ displayed a significantly reduced crawling speed compared to wild-type *w*^*1118*^ (**Fig 4B and 4C**) suggesting that the mutation results in a potential fitness cost. Physical fitness costs caused by point mutations were also reported in other species of insect pests [43,54–58]. For example, *D*. *melanogaster* bearing the homozygous R81T in the nicotinic acetylcholine receptor β1 subunit showed an increased tolerance to neonicotinoid insecticides with a dramatic decrease in fertility, locomotivity and longevity [56].

In conclusion, the G3’_TMD3_ residue in RDL was identified *in silico* and *in vitro* as being important for the interaction of arthropod GABAR with broflanilide or fluralaner and its role in the sensitivity of *D*. *melanogaster* to these insecticides was verified *in vivo* using the CRISPR/Cas9 system. Our results showed that: 1) both broflanilide and fluralaner act on the G3’_TMD3_ residue of the RDL subunit; 2) a heterozygous mutation at G3’_TMD3_ is unlikely to lead to resistance to broflanilide and fluralaner; 3) a homozygous G3’_TMD3_ mutation prevented development beyond the larval stage. Our results could help systematically define the interaction of meta-diamides and isoxazolines with their molecular targets as well as further understand possible routes to resistance to these insecticides. Also, our findings may aid in the design and modification of novel insecticides, important for our continual management of agricultural and sanitary pests.

## Materials and methods

### Ethics statement

The use of *X*. *laevis* in the present study strictly followed the ethics of the China (GB/T 35892–2018) and Nanjing Agricultural University guidelines (https://dongwu.njau.edu.cn/info/1003/1192.htm) for the protection of animal welfare.

### Chemicals

Fluralaner (purity ≥ 99%) was purified from Bravecto [59]. Fipronil (purity ≥ 96%) was provided by J & K Scientific (China) Ltd. (Beijing, CHN). Avermectin (purity ≥ 92%) was provided by Jiangsu Fengyuan Bio-Engineering Co., Ltd. (Yancheng, Jiangsu Province, CHN). Broflanilide (purity ≥ 98.67%) was provided by Mitsui Chemicals Agro, Inc. (Tokyo, JPN). Reagents and solvents used in the present study were obtained from commercial suppliers.

### Insect strains and mouse mRNA

Both *D*. *melanogaster* strains *w*^*1118*^ and nos.Cas9 (stock #54591 at Bloomington *Drosophila* Stock Center) were used as the G_0_ generation for genome-modification. The balancer strain (*w*^*1118*^; TM2 *Ubx*^*130*^/TM6B *Tb*^*1*^; stock #FWB00002 at Fungene Biotech) was used for balancing. The *w*^*1118*^; dIRE1^Δ^/TM3 *Sb GFP* strain (stock #293 at Shanghai Institute of Biochemistry and Cell Biology) was used for replacing the balancer chromosome. The deficiency strain (*w*^*1118*^; Df(3L)BSC170/TM6B *Tb*^*1*^; stock #9561 at Bloomington *Drosophila* Stock Center) was used for the complementation experiment. All the flies were cultured on standard fly diet at 25°C, relative humidity of 50%-60% and a 12:12 h (L: D) photoperiod. The mouse *M*. *musculus* mRNA was kindly provided by Dr. Hui-Xing Lin (Nanjing Agricultural University).

### Prediction and site-directed mutagenesis of mutation sites

An alignment of amino acid sequences of GABAR subunits from arthropods and vertebrates was constructed using MEGA 7.0 software [60] and then manually adjusted using GeneDoc (version 2.6.002) software (Pittsburgh Supercomputing Center; http://www.psc.edu/biomed/genedoc/). Thirty five amino acid residues that differed between arthropod and vertebrate sequences were selected (**S3 Fig and S1 Table**). The *C*. *suppressalis* RDL homology model was constructed by SWISS-MODEL (https://swissmodel.expasy.org/) using the human GABA_A_R-β3 homopentamer (PDB code: 4COF) as template. The constructed model was evaluated by the online programs of PROCHECK [61,62] and ProSA-web [63]. The structure of fluralaner was built using SYBYL-X 2.1 software (Tripos Inc. St. Louis, CA) running on a Windows 7 workstation, and was then docked onto the *Cs*RDL model using Surflex-Dock module of SYBYL-X. 2.1. The potential docking site(s) were generated using the residue-based mode of Surflex-Dock with other parameters selected as default. Different single- or double-point mutations (**S2 Table**) of *Cs*RDL were generated using the Mutate Monomers module of SYBYL-X 2.1 and then the mutant models were minimized under the Tripos force field with MMFF94 charges by the Powell method with a gradient convergence criterion of 0.005 kcal/mol Å. Mutant amino acid residues, which lead to significant changes in binding affinity of fluralaner, were selected for further analysis.

### Heterologous expression of GABARs in *X*. *laevis* oocytes

All predicted binding sites were mutated using specific primers (**S11 Table**) and the Fast Mutagenesis System (TransGen Biotech, Beijing, CHN) as reported previously [64]. GABAR subunit coding sequences were cloned into the pGH19 plasmid for expression in *X*. *laevis* oocytes. Procedures for oocyte preparation and cRNA injection into oocytes were identical to those described previously [65]. The ratio of the two injected subunits in the heteromeric *Mm*α1β2 and *Mm*α1β2-M3’G_TMD3_ GABARs was 1: 1.

### Electrophysiological assays

Two-electrode voltage-clamp electrophysiology was performed and recorded on the Axoclamp 900A Microelectrode Amplifier platform (Molecular Devices, San Jose, CA) at a holding potential of -60 mV as previously described [65,66]. The Axon Digidata 1440A Data Acquisition System (Molecular Devices) was used to record the current signals. Oocytes were placed in a recording chamber (RC-3Z, Warner Instruments, Hamden, CT) with standard oocytes saline (SOS) medium [64] at a perfusion speed of 8–10 mL/min. Electrophysiological assays were performed at 20°C. GABA was dissolved in SOS medium and applied to oocytes for 5 s at intervals of 85 s. Concentration-response curves were obtained by sequential applications of increasing concentrations of GABA. The median effective concentration (EC_50_) was calculated with GraphPad Prism 5 (GraphPad Software, Inc., San Diego, CA). For antagonist assays, insecticides were first dissolved in dimethyl sulfoxide (DMSO) then diluted with SOS medium to a final DMSO concentration less than 0.1% (v/v), which did not affect a response from oocytes. Following two successive control applications of the GABA (EC_50_), the insecticide solution was perfused alone for 85 s before GABA (EC_50_) was co-applied with insecticide for 5 s, and the co-application was repeated 4–5 times at 85 s intervals to obtain the maximum constant inhibition. The values of EC_50_, median inhibitory concentration (IC_50_) and the scatter plot were determined from the mean of 3–15 replicates using non-linear regression analysis on GraphPad Prism 5. Two values of EC_50_ or IC_50_ were considered significantly different if their 95% confidence intervals (CIs) did not overlap [67].

### Bioassays

The bioassay method for *D*. *melanogaster* adults was based on ffrench-Constant and Roush (1991) [16] with slight modifications. Insecticide was coated internally on the inside of glass vials (diameter × height, 20 mm × 80 mm) by applying 150 μL of acetone containing various concentrations of each insecticide, and rolling the vials until the acetone evaporated. Fifteen female flies (1–3 days post-eclosion) were transferred into the vial as a replicate, and each vial was plugged with absorbent cotton (~ 0.5 g) soaked with 5% sucrose (4.0 mL). The mortality was recorded at 72 h for avermectin and at 24 h for the other insecticides after treatment. For these assays, each concentration was replicated three times and acetone treatment was used as a control. The median lethal concentration (LC_50_) was calculated by probit analysis using SPSS 17.0 (SPSS Inc., Chicago, IL). A χ^2^ test was used to assess how well the individual LC_50_ values agreed with the calculated linear regression lines.

For *D*. *melanogaster* larvae bioassays, insecticide was dissolved in acetone and then mixed with the fly diet (based on corn powder, white sugar, yeast and agar) to obtain a series of concentrations. A replicate consisting of fifteen newly-hatched (less than 1 h) larvae were placed individually in single chambers of a 48-well culture plate containing 0.15 g of prepared standard fly diet. After 24 h, the mortality was calculated. Each concentration was studied in triplicate and 1% (v/w) acetone treatment was used as a control. The LC_50_ value was calculated as for adult bioassays.

### Genomic engineering strategy

The CRISPR/Cas9 system was used to generate point mutations altering the G3’_TMD3_ residue in the *Dm*RDL subunit. First, a 447-bp genomic region encompassing the codon for G3’_TMD3_ was sequenced to identify possible SNPs. Potential CRISPR targets in the region of interest were identified using the online platform CRISPR Optimal Target Finder (http://tools.flycrispr.molbio.wisc.edu/targetFinder/). Two target sequences (gRNA1 and gRNA2, **S6 Fig**) without predicted off-target sites were selected for genomic editing.

To generate the G3’M_TMD3_ or G3’S_TMD3_ mutations, Cas9 mRNA, gRNAs and donor plasmid were prepared and gently mixed before injection. Briefly, Cas9 mRNA was transcribed from the linearized plasmid MLM3613 (Addgene #42251) using the mMESSAGE mMACHINE T7 Transcription Kit (Ambion, Carlsbad, CA), polyadenylated with the *E*. *coli* Poly(A) polymerase Kit (New England Biolabs, MA, ENG) then purified with the RNeasy Mini Kit (QIAGEN, Duesseldorf, GER). *In vitro* transcriptions (IVT) of gRNAs were performed following the protocol of Bassett et al. (2013) [36] where templates were generated by annealing two DNA oligonucleotides (*Dmrdl*-gRNA1-F/R and *Dmrdl*-gRNA2-F/R, **S11 Table**). Then RNA was generated by IVT using the T7 RiboMAX Express Large Scale RNA Production System (Promega Corporation, Madison, WI) before being purified by phenol-chloroform extraction and isopropanol precipitation. The pBluescript SK (-) donor plasmids (pBS-G3M, pBS-G3S), which contained two ~1,000-bp homology arms flanking the targeted genomic region with certain modifications, was constructed *de novo* using primers (*Dmrdl*-G3-5-F/R, *Dmrdl*-G3M-3-F/R, *Dmrdl*-G3S-3-F/R, pBSK-s-F/R, **S11 Table**) with the Gibson Assembly Kit (New England Biolabs) for HDR.

To generate the G3’Q_TMD3_ mutation, specific gRNA-expressing plasmid and donor plasmid were constructed. For the gRNA-expressing plasmid, a DNA fragment with two fused gRNA sequences was amplified using primers (pCFD4-gRNAs-F/R, **S11 Table**) and the pCFD4 plasmid (Addgene#49411) backbone as template. Then the modified fragment was recombined into *Bbs* I-digested pCFD4 vector with the ClonExpress II One Step Cloning kit (Vazyme, Nanjing, CHN). The donor plasmid pBS-G3Q was constructed using the Fast Mutagenesis System (Transgen) and the pBS-G3M plasmid as template with primers G3QDonor-F/R (**S11 Table**).

### Purification and amplification of genomic DNA

Genomic DNA was extracted and purified from whole *D*. *melanogaster* using DNAiso Reagent (TaKaRa, Beijing, CHN). Using genomic DNA as template, PCR amplification with primer pairs (rdl335det-F/R, **S11 Table**) was typically performed with 2 × Rapid Taq Master Mix (Vazyme). The conditions used were 95°C for 3min, followed by 35 cycles of denaturation at 95°C for 15 s, annealing at 54°C for 15 s, and extension at 72°C for 30 s, ending with a final extension step at 72°C for 5 min.

### Generation and identification of genome-modified *D*. *melanogaster* lines

To generate G3’M_TMD3_ and G3’S_TMD3_ strains, embryo injection was performed according to standard protocols [41]. The mixture containing Cas9 mRNA (500 ng/ μL), gRNAs (250 ng/ μL of each) and donor plasmid (500 ng/ μL) were co-injected into embryos. The injected G_0_ adult flies were individually crossed to the balancer strain (*w*^*1118*^; TM2 *Ubx*^*130*^/TM6B *Tb*^*1*^) and analyzed for HDR alleles (**S7 Fig**). G_1_ adult flies from positive lines with a TM2 *Ubx*^*130*^ allele were individually crossed with the balancer strain and screened. Subsequently, the G_2_ adult flies with both HDR and TM2 *Ubx*^*130*^ alleles were self-crossed to generate heterozygous mutant strains (G_3_). Both of the mutant strains were verified by sequencing and maintained by self-crossing.

To generate G3’Q_TMD3_ strains, a mixture of gRNA-expressing plasmid (200 ng/μL) and donor plasmid (150 ng/μL) were injected into embryos of nos.Cas9. The G_0nos_ adult flies were individually crossed with nos.Cas9 flies. Subsequently, screening of G_1nos_ flies were performed by extracting DNA from sets of ~15 individuals (adult flies) per vial. Once the HDR alleles were detected in these G_1nos_ flies, other male flies from the same positive lines were individually crossed with females of the balancer strain (*w*^*1118*^; TM2 *Ubx*^*130*^/TM6B *Tb*^*1*^) (**S7 Fig**). When G_2nos_ progeny emerged, male flies with a TM2 *Ubx*^*130*^ allele were screened after individually crossing with female balancer flies, and the lines arising from mutant G_2nos_ male flies were retained. By this step, all nos.Cas9 in the X chromosome were replaced with *w*^*1118*^ background. In the G_3nos_ generation, adult flies with both HDR and TM2 *Ubx*^*130*^ alleles were self-crossed to generate a mutant strain (G_4nos_), which was verified by genomic DNA sequencing.

### Complementation experiment

To investigate if the observed lethality is genetically linked to the *Rdl* gene region, a complementation experiment was performed by crossing heterozygous mutant flies to the deficiency strain (*w*^*1118*^; Df(3L)BSC170/TM6B *Tb*^*1*^), which carries a deletion of a genomic region (3L:9058052; 3L:9183797) containing *Rdl*. For each strain, 10 crosses were set, and the genotypes of progeny were determined after emerging by the balancer-associated phenotypes.

### Temporal tracking of lethality in homozygous mutations

Heterozygous mutant flies were crossed with the dIRE1^Δ^/TM3 *Sb GFP* strain to replace the balancer TM2 *Ubx*^*130*^ with TM3 *Sb* GFP. Female adults of G3’M_TMD3_/TM3 *Sb GFP*, G3’S_TMD3_/TM3 *Sb GFP* or G3’Q_TMD3_/TM3 *Sb GFP* strains were induced to oviposit on a juice plate for 2 h. Embryos were collected and transferred onto an agar plate (1.5%, w/w) for hatching. After 24 h, twenty homozygous mutants as one repetition were selected from newly-hatched larvae using the SMZ25 stereomicroscope (Nikon Corporation, Tokyo, JPN) and placed individually in single chambers of a 48-well culture plate containing 0.3 g standard fly diet. Then the survival rate of flies was recorded every 24 h until the seventh day. Three repetitions were performed for each genotype, and the *w*^*1118*^ strain was used as the control. Data were analyzed with GraphPad Prism 5 using the Log-rank test for trend and the Mantel-Cox test. Significant difference was determined by *P* values: ns indicates *P* > 0.05, * indicates *P* < 0.05, *** indicates *P* < 0.001.

### Crawling assay

Larvae homozygous for G3’M_TMD3_, G3’S_TMD3_ or G3’Q_TMD3_ were selected for the crawling assay. Four larvae as one repetition were placed on an agar plate (1.5%, w/w) and movement was recorded using a stereomicroscope combined with the Mshot Digital Imaging System (Mshot Optoelectronics Technology Co., Ltd., Guangzhou, Guangdong Province, CHN) for 2 min. Recorded videos were converted to AVI format using Format Factory (Free Time, Shanghai, CHN), and analyzed with ImageJ (National Institutes of Health, Bethesda, MD) [68, 69]. Five repetitions were performed for each genotype, and the *w*^*1118*^ strain was used as the control. Crawling speed was analyzed with Student’s *t*-test using SPSS 17.0 (SPSS Inc.). Significant difference was determined by *P* values: *** indicates *P* < 0.001.

## Supporting information

S1 Video Characterization of crawling in *w*^*1118*^ and homozygous mutant flies. The content was filmed at 3.33 fps, played at 30.00 fps and 30-times original speed.
(MOV)

S1 Fig Structures of insecticides acting as noncompetitive antagonist and/or allosteric modulators of GABARs. The year of discovery or first introduction is included.
(PDF)

S2 Fig Structure of the *Dm*RDL subunit and sequence alignment of the TMD3 from several insect species. **(A)** Structure model of an RDL subunit built by Modeller 10.3 based on a *C*. *elegans* GluCl structure (PDB ID: 3RHW). The G3’ (G335) residue in TMD3 is labeled in green. **(B)** Amino acid sequence alignment of TMD3 of RDL. ***Dm***: *Drosophila melanogaster*, ***Md***: *Musca domestica*, ***Lc***: *Lucilia cuprina*, ***Am***: *Apis mellifera*, ***Sl***: *Spodoptera litura*, ***Bm***: *Bombyx mori*. The GenBank accession numbers of the amino acid sequences are *Dm*RDL (AAA2856), *Md*RDL (AB177547), *Lc*RDL (AAB81966), *Am*RDL (AJE68941), *Sl*RDL (BAW87784) and *Bm*RDL (NP_001182630).
(PDF)

S3 Fig Comparison of amino acid sequences of GABAR subunits from different species. (PDF)

S4 Fig Alignment of the third transmembrane domain (TMD3) and the flanking amino acid sequences of GABAR subunits from mammals and arthropods. The subunit-specific number is given at the left for the first residue of each aligned sequence and the index numbers for positioning in TMD3 are shown at the bottom. To facilitate the alignment of GABAR subunits from different species, the nomenclature, which is used for TMD2 of insect RDL, was employed as well in this study. Therefore, the first amino acid residue preceding TMD3 is designated as “0”. ***Hs***: *Homo sapiens*, ***Mm***: *Mus musculus*, ***Cs***: *Chilo suppressalis*, ***Ls***: *Laodelphax striatellus*, ***Am***: *Apis mellifera*, ***Dm***: *Drosophila melanogaster*, ***Tu***: *Tetranychus urticae*. The GenBank accession numbers of the amino acid sequences are *Hs*α1 (NP_001121120.1), *Mm*α1 (NP_034380.1), *Hs*β2 (NP_000804.1), *Mm*β2 (NP_001334243.1), *Hs*β3 (NP_068712.1), *Mm*β3 (NP_001033790.1), *Hs*γ2 (AAH74795.1), *Mm*γ2 (NP_032099.1), *Cs*RDL (ASY91962.1), *Ls*RDL (BAF31884.1), *Am*RDL (ANC68177.1), *Dm*RDL (NP_523991.2) and *Tu*RDL (BAJ41377.1).
(PDF)

S5 Fig Current traces showing responses of wild-type or G3’M_TMD3_ *Cs*RDL to GABA and fluralaner. **(A)** and **(B)**, Representative concentration-dependent current traces of wild-type or G3’M_TMD3_ *Cs*RDL induced by GABA. **(C)** and **(D)**, Representative current traces of inhibition of GABA-induced currents by fluralaner applied to wild-type or G3’M_TMD3_ *Cs*RDL.
(PDF)

S6 Fig Genomic editing strategy of CRISPR/Cas9 on *Drosophila melanogaster*. A 2114-bp nucleotide sequence (chromosome 3L: 9163248–9165362) was used as the homology region in the donor plasmid. Exon 7 of the *Rdl* gene is marked with yellow rectangles. Dark blue rectangles indicate the gRNA targeted sequences while grey rectangles indicate the corresponding protospacer adjacent motif (PAM) triplets. The G3’_TMD3_ codon is marked with a red asterisk and bases for synonymous mutations are shown in light blue. The rdl335det-F/R primers used for sequencing target mutations is shown in purple half arrows.
(PDF)

S7 Fig Crossing procedures for the generation of genome-modified *Drosophila* strains. **(A)** Procedure for the generation of G3’M/S_TMD3_ mutant strains. HDR indicates a G3’M_TMD3_ or G3’S_TMD3_ allele. **(B)** Procedure for the generation of the G3’Q_TMD3_ strain. Background in the X chromosome is nos.Cas9, and HDR indicates a G3’Q_TMD3_ mutant allele.
(PDF)

S1 Table Prediction of potential amino acid residues in *Cs*RDL interacting with fluralaner. (PDF)

S2 Table Potencies of GABA on *X. laevis* oocytes injected with CsRDL cRNA. (PDF)

S3 Table Inhibition of GABA-induced currents by fluralaner in *X*. *laevis* oocytes injected with *Cs*RDL cRNA. (PDF)

S4 Table Inhibition of GABA-induced currents by avermectin and fipronil in *X*. *laevis* oocytes injected with G3’M_TMD3_ *Cs*RDL cRNAs. (PDF)

S5 Table Potencies of GABA and fluralaner on wild-type or G3’M_TMD3_ RDL cRNA from different species expressed in *X*. *laevis* oocytes. (PDF)

S6 Table Potencies of GABA on heteromeric *Mm*α1β2 or *Mm*α1β2-M3’G_TMD3_ receptors expressed in *X*. *laevis* oocytes. (PDF)

S7 Table Inhibition by fluralaner on GABA-induced currents in *X*. *laevis* oocytes injected with *Mm*α1β2 or *Mm*α1β2-M3’G_TMD3_. (PDF)

S8 Table Toxicity of four insecticides to *w*^*1118*^ adults and heterozygous adults bearing G3’_TMD3_ mutations. (PDF)

S9 Table Survival rate of larvae of *w*^*1118*^ and homozygous for G3’_TMD3_ mutations. (PDF)

S10 Table Crawling speed of larvae of *w*^*1118*^ and homozygous for G3’_TMD3_ mutations. (PDF)

S11 Table Primers used in this study. (PDF)

S1 Note Amino acid residues of RDL subunits. Note, the amino acids marked as yellow are the TMDs, and all the potential amino acid residues predicted to interact with fluralaner are shown in red, while the G3’_TMD3_ (wild-type) residue is marked in green and the M3’_TMD3_ (mutant) residue is marked in blue.
(PDF)
