# Supplementary figures and images for: G3’MTMD3 in the insect GABA receptor subunit, RDL, confers resistance to broflanilide and fluralaner
Source: PLoS Genet. 2023 Jun 29;19(6):e1010814. doi: 10.1371/journal.pgen.1010814 (PMC10337980; doi:10.1371/journal.pgen.1010814)

**S1 Fig**

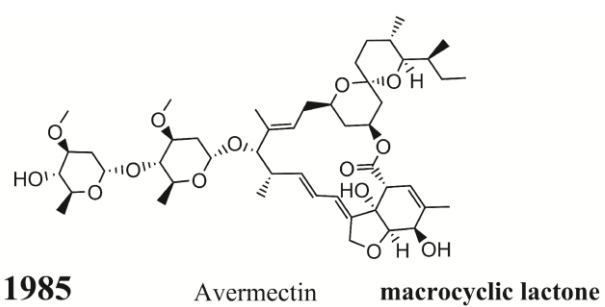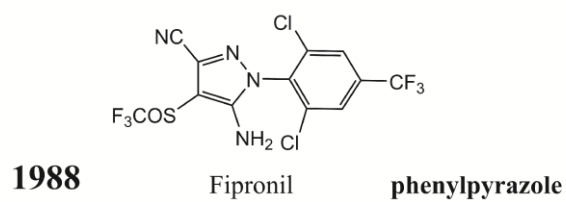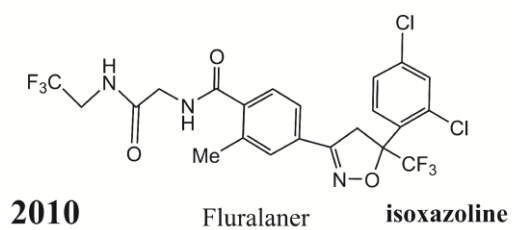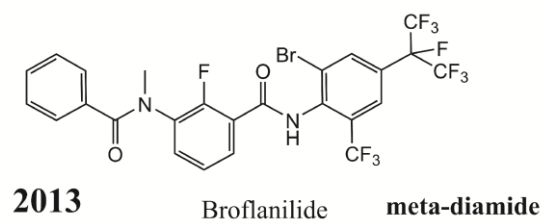

Supplement: S1 Fig — The year of discovery or first introduction is included. (PDF) [file pgen.1010814.s002.pdf]

S2 Fig

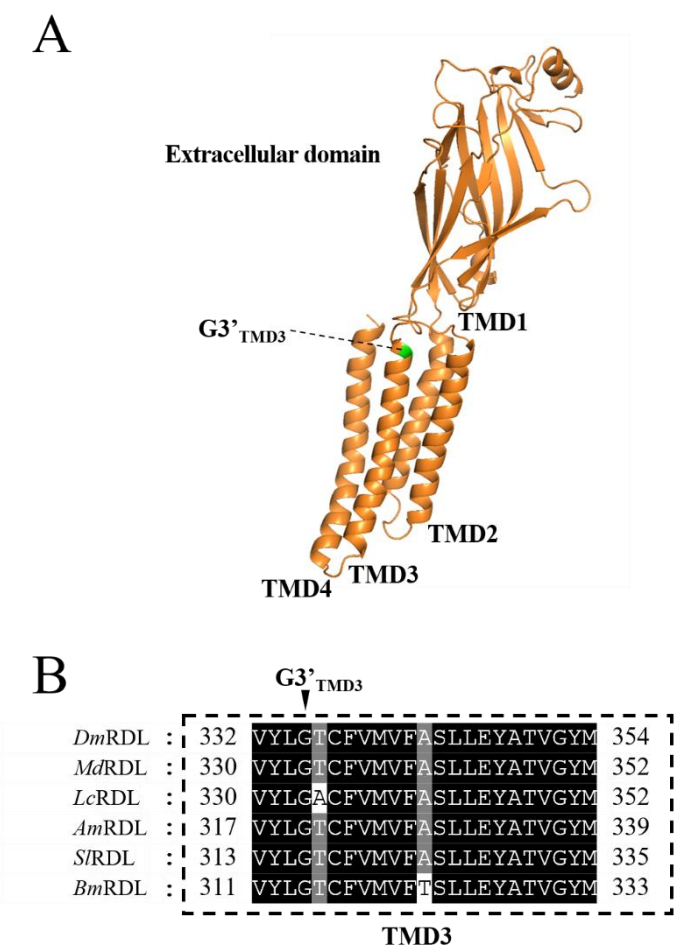

Supplement: S2 Fig — (A) Structure model of an RDL subunit built by Modeller 10.3 based on a C. elegans GluCl structure (PDB ID: 3RHW). The G3’ (G335) residue in TMD3 is labeled in green. (B) Amino acid sequence alignment of TMD3 of RDL. Dm: Drosophila melanogaster, Md: Musca domestica, Lc: Lucilia cuprina, Am: Apis mellifera, Sl: Spodoptera litura, Bm: Bombyx mori. The GenBank accession numbers of the amino acid sequences are DmRDL (AAA2856), MdRDL (AB177547), LcRDL (AAB81966), AmRDL (AJE68941), SlRDL (BAW87784) and BmRDL (NP_001182630). (PDF) [file pgen.1010814.s003.pdf]

# S4 Fig

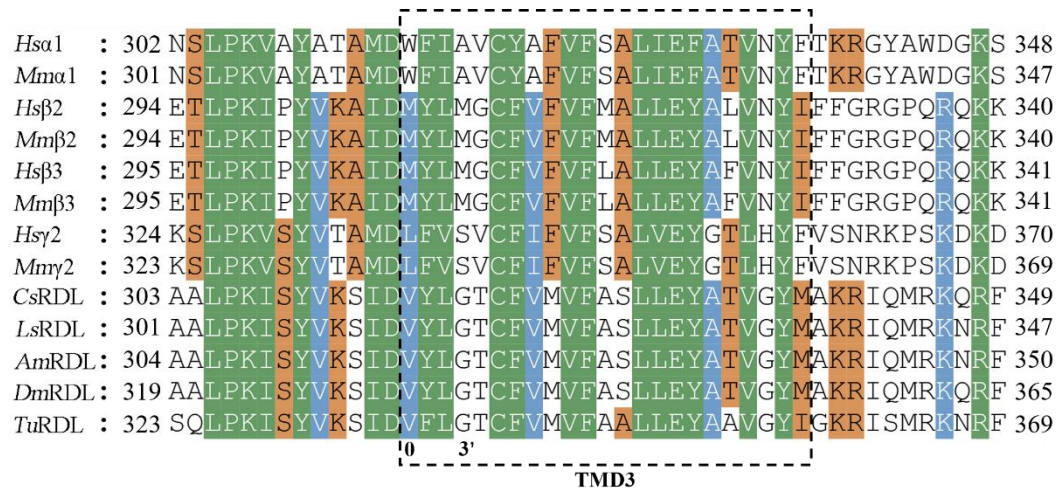

Supplement: S4 Fig — The subunit-specific number is given at the left for the first residue of each aligned sequence and the index numbers for positioning in TMD3 are shown at the bottom. To facilitate the alignment of GABAR subunits from different species, the nomenclature, which is used for TMD2 of insect RDL, was employed as well in this study. Therefore, the first amino acid residue preceding TMD3 is designated as “0”. Hs: Homo sapiens, Mm: Mus musculus, Cs: Chilo suppressalis, Ls: Laodelphax striatellus, Am: Apis mellifera, Dm: Drosophila melanogaster, Tu: Tetranychus urticae. The GenBank accession numbers of the amino acid sequences are Hsα1 (NP_001121120.1), Mmα1 (NP_034380.1), Hsβ2 (NP_000804.1), Mmβ2 (NP_001334243.1), Hsβ3 (NP_068712.1), Mmβ3 (NP_001033790.1), Hsγ2 (AAH74795.1), Mmγ2 (NP_032099.1), CsRDL (ASY91962.1), LsRDL (BAF31884.1), AmRDL (ANC68177.1), DmRDL (NP_523991.2) and TuRDL (BAJ41377.1). (PDF) [file pgen.1010814.s005.pdf]

**S5 Fig**

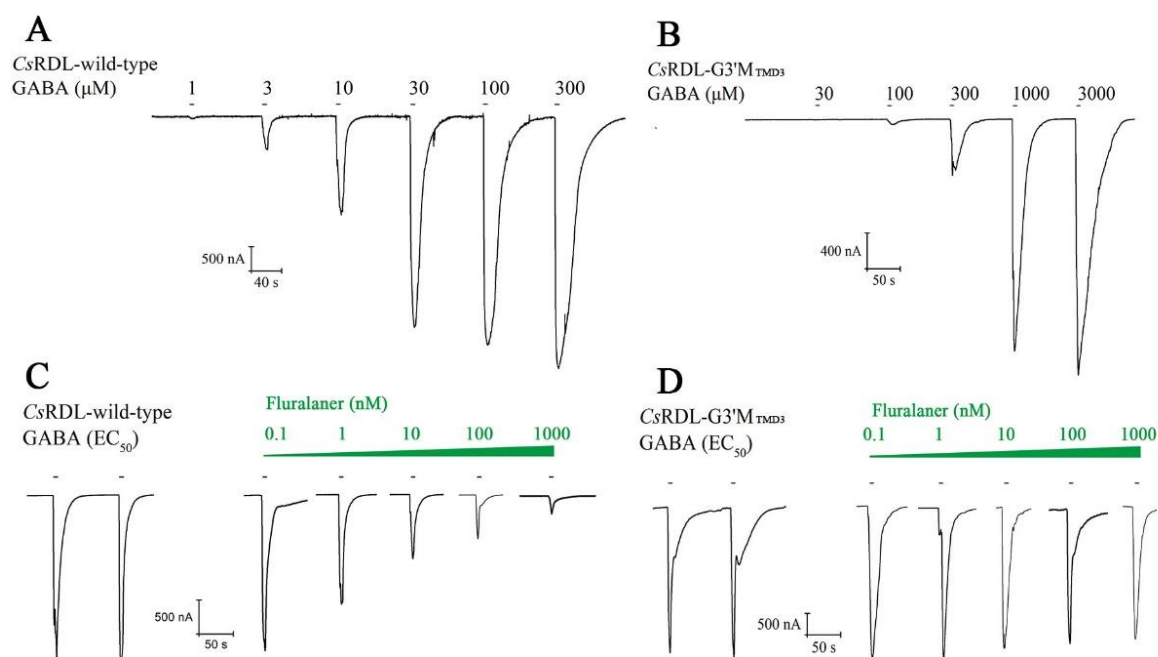

Supplement: S5 Fig — (A) and (B), Representative concentration-dependent current traces of wild-type or G3’MTMD3 CsRDL induced by GABA. (C) and (D), Representative current traces of inhibition of GABA-induced currents by fluralaner applied to wild-type or G3’MTMD3 CsRDL. (PDF) [file pgen.1010814.s006.pdf]

## S6 Fig

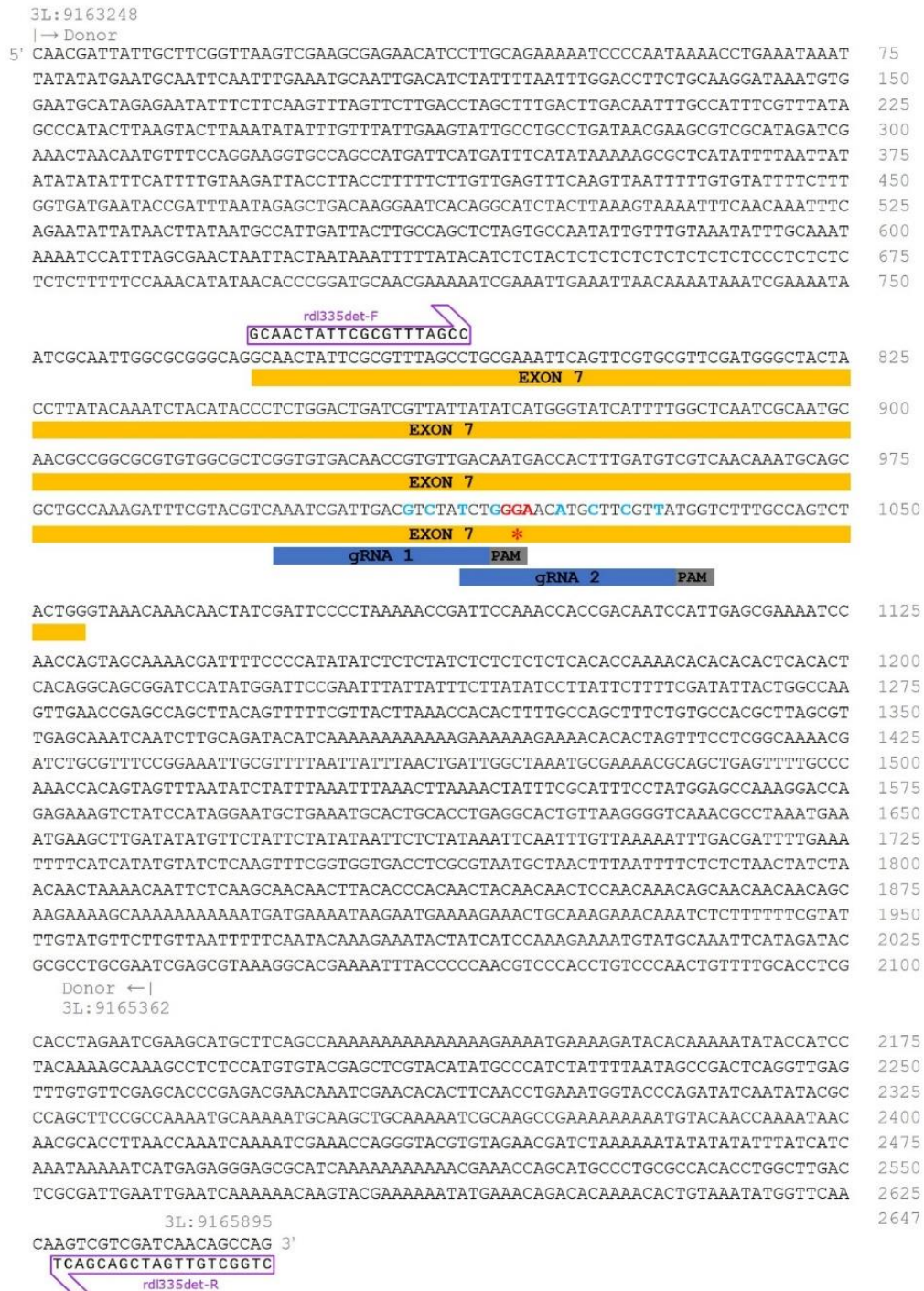

Supplement: S6 Fig — A 2114-bp nucleotide sequence (chromosome 3L: 9163248–9165362) was used as the homology region in the donor plasmid. Exon 7 of the Rdl gene is marked with yellow rectangles. Dark blue rectangles indicate the gRNA targeted sequences while grey rectangles indicate the corresponding protospacer adjacent motif (PAM) triplets. The G3’TMD3 codon is marked with a red asterisk and bases for synonymous mutations are shown in light blue. The rdl335det-F/R primers used for sequencing target mutations is shown in purple half arrows. (PDF) [file pgen.1010814.s007.pdf]

**S7 Fig**

**A**

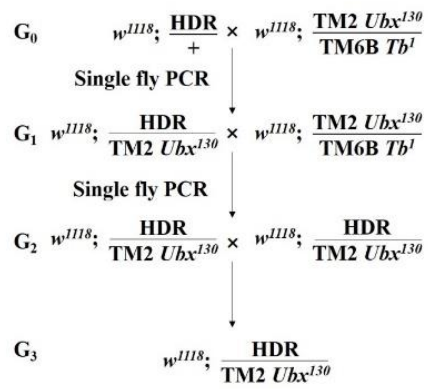

**B**

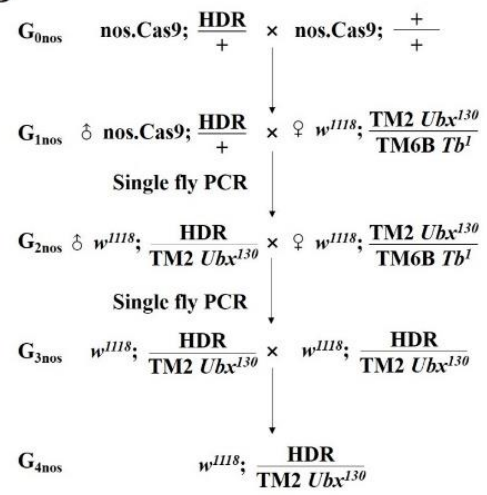

Supplement: S7 Fig — (A) Procedure for the generation of G3’M/STMD3 mutant strains. HDR indicates a G3’MTMD3 or G3’STMD3 allele. (B) Procedure for the generation of the G3’QTMD3 strain. Background in the X chromosome is nos.Cas9, and HDR indicates a G3’QTMD3 mutant allele. (PDF) [file pgen.1010814.s008.pdf]
