# Supplementary material for: G3’MTMD3 in the insect GABA receptor subunit, RDL, confers resistance to broflanilide and fluralaner
Source: PLoS Genet. 2023 Jun 29;19(6):e1010814. doi: 10.1371/journal.pgen.1010814 (PMC10337980; doi:10.1371/journal.pgen.1010814)
Supplement: S3 Fig — (PDF) [file pgen.1010814.s004.pdf]

S3 Fig

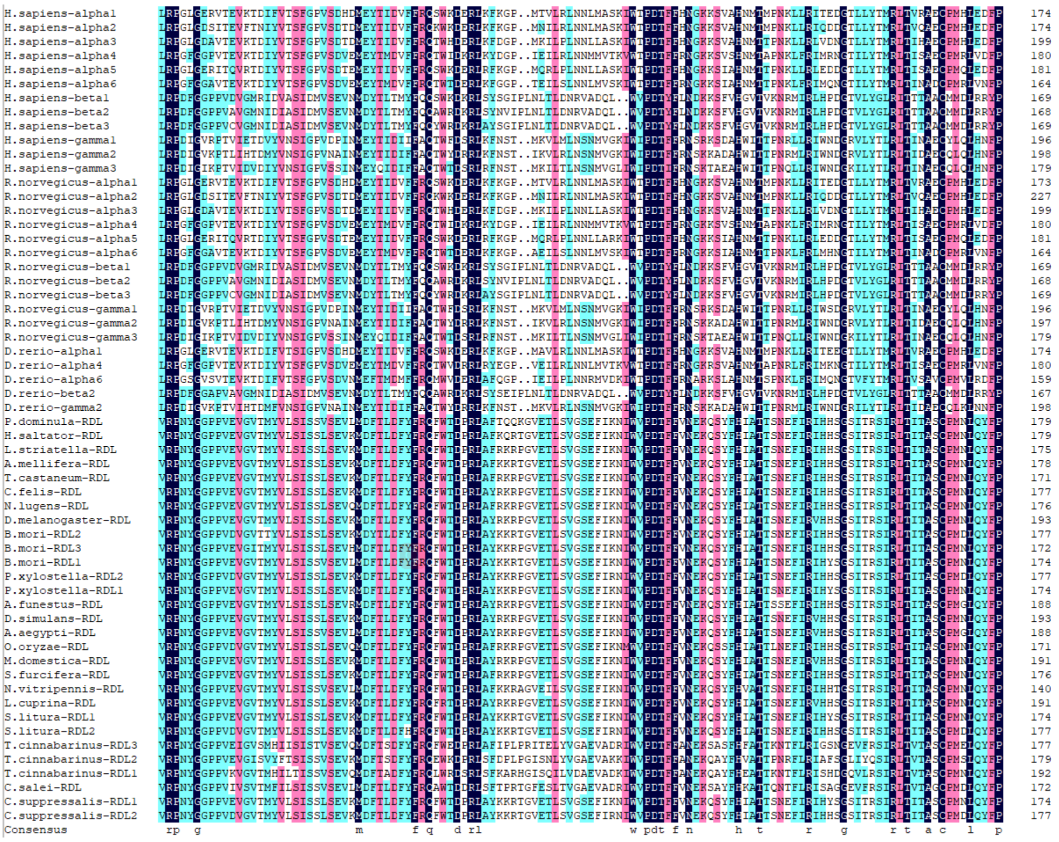

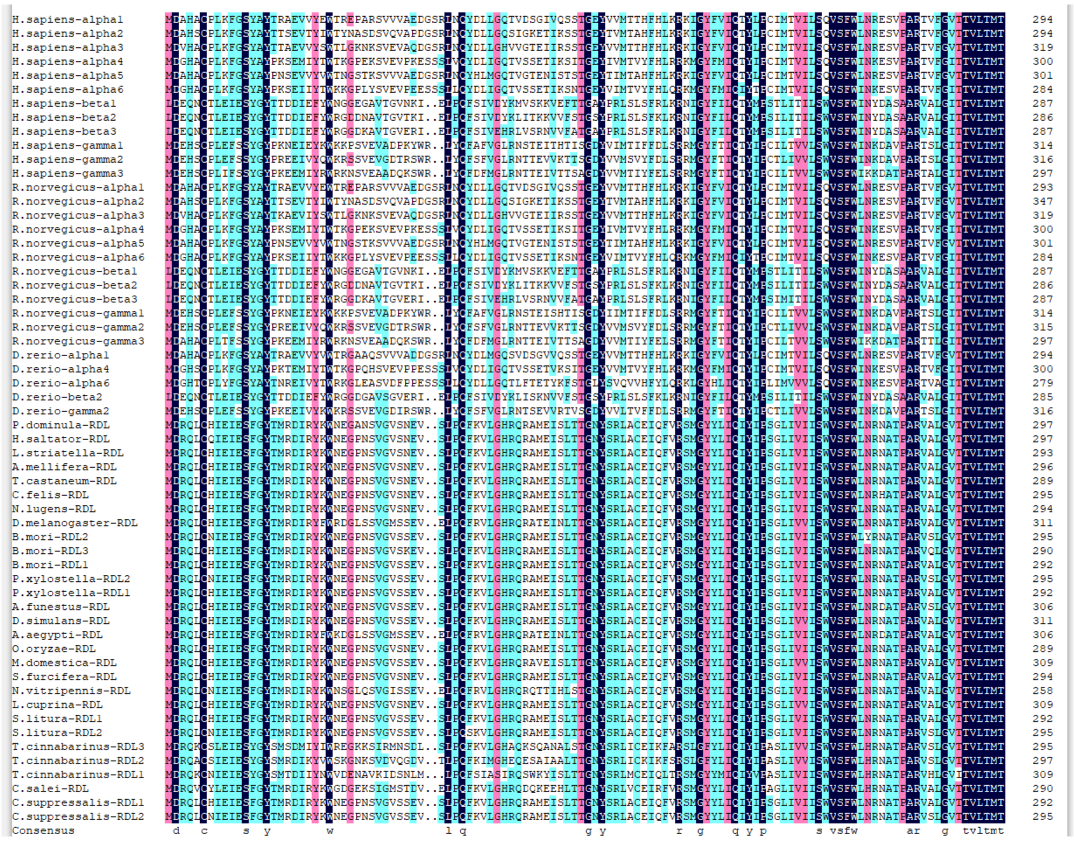

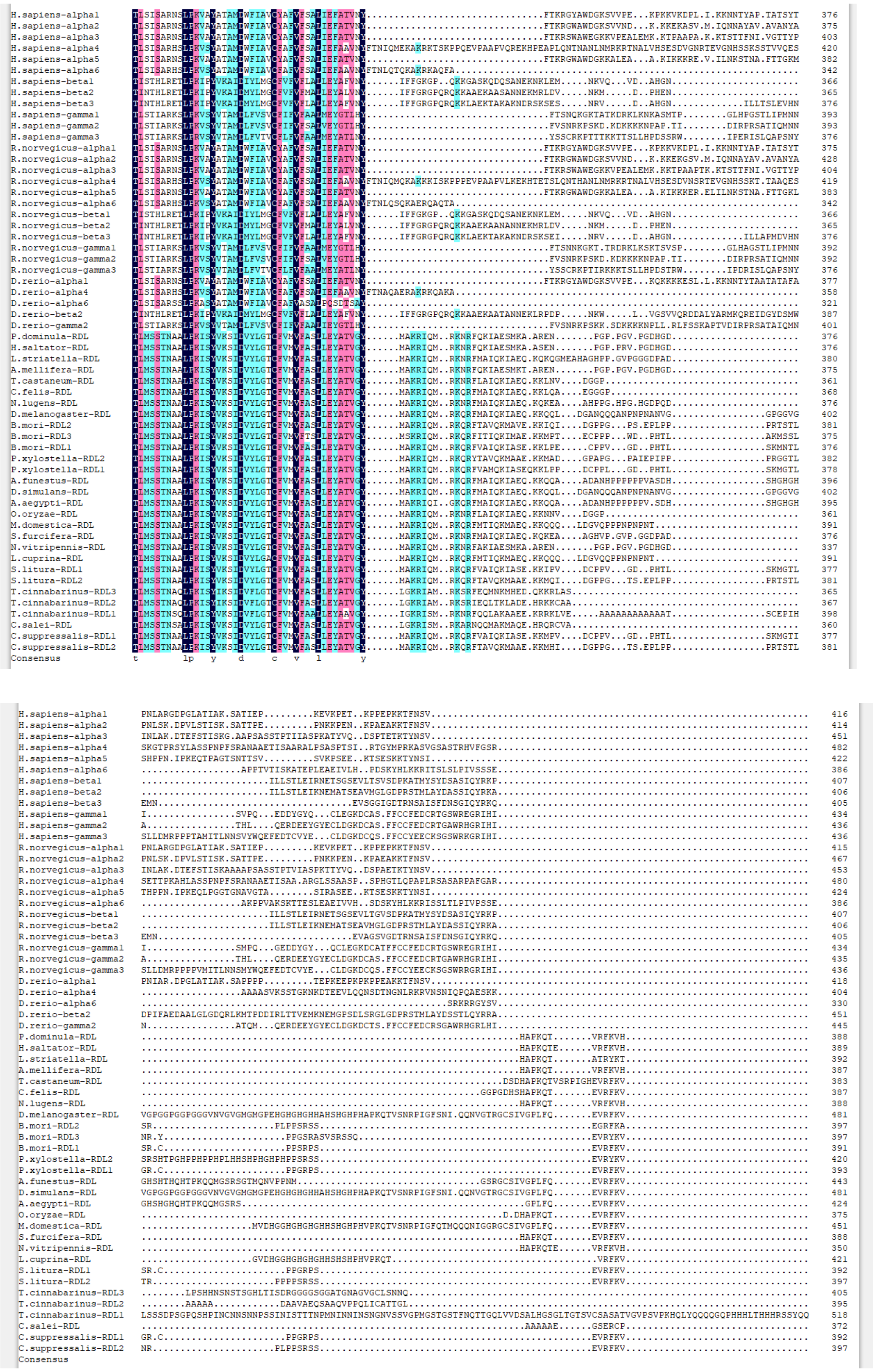

```
H.sapiens-alpha1        ........................................................................................................................  416
H.sapiens-alpha2        ........................................................................................................................  414
H.sapiens-alpha3        ........................................................................................................................  451
H.sapiens-alpha4        ........................................................................................................................  482
H.sapiens-alpha5        ........................................................................................................................  422
H.sapiens-alpha6        ........................................................................................................................  386
H.sapiens-beta1         ........................................................................................................................  407
H.sapiens-beta2         ........................................................................................................................  406
H.sapiens-beta3         ........................................................................................................................  405
H.sapiens-gamma1        ........................................................................................................................  434
H.sapiens-gamma2        ........................................................................................................................  436
H.sapiens-gamma3        ........................................................................................................................  436
R.norvegicus-alpha1     ........................................................................................................................  415
R.norvegicus-alpha2     ........................................................................................................................  467
R.norvegicus-alpha3     ........................................................................................................................  453
R.norvegicus-alpha4     ........................................................................................................................  480
R.norvegicus-alpha5     ........................................................................................................................  424
R.norvegicus-alpha6     ........................................................................................................................  386
R.norvegicus-beta1      ........................................................................................................................  407
R.norvegicus-beta2      ........................................................................................................................  406
R.norvegicus-beta3      ........................................................................................................................  405
R.norvegicus-gamma1     ........................................................................................................................  434
R.norvegicus-gamma2     ........................................................................................................................  435
R.norvegicus-gamma3     ........................................................................................................................  436
D.rerio-alpha1          ........................................................................................................................  418
D.rerio-alpha4          ........................................................................................................................  404
D.rerio-alpha6          ........................................................................................................................  330
D.rerio-beta2           ........................................................................................................................  451
D.rerio-gamma2          ........................................................................................................................  445
P.dominula-RDL          ........................................................................................................................  388
H.saltator-RDL          ........................................................................................................................  389
L.striatella-RDL        ........................................................................................................................  392
A.mellifera-RDL         ........................................................................................................................  387
T.castaneum-RDL         ........................................................................................................................  383
C.felis-RDL             ........................................................................................................................  387
N.lugens-RDL            ........................................................................................................................  388
D.melanogaster-RDL      ........................................................................................................................  481
B.mori-RDL2             ........................................................................................................................  397
B.mori-RDL3             ........................................................................................................................  397
B.mori-RDL1             ........................................................................................................................  391
P.xylostella-RDL2       ........................................................................................................................  420
P.xylostella-RDL1       ........................................................................................................................  393
A.funestus-RDL          ........................................................................................................................  443
D.simulans-RDL          ........................................................................................................................  481
A.aegypti-RDL           ........................................................................................................................  424
O.oryzae-RDL            ........................................................................................................................  375
M.domestica-RDL         ........................................................................................................................  451
S.furcifera-RDL         ........................................................................................................................  388
N.vitripennis-RDL       ........................................................................................................................  350
L.cuprina-RDL           ........................................................................................................................  421
S.litura-RDL1           ........................................................................................................................  392
S.litura-RDL2           ........................................................................................................................  397
T.cinnabarinus-RDL3     ........................................................................................................................  405
T.cinnabarinus-RDL2     ........................................................................................................................  395
T.cinnabarinus-RDL1     HHQHLQQQLQQQQQQHQSIGTLTSVSNLGVGLSVPSNLVTYDFSGNGLSGGSGNLMMTTNPEMINCSLGPGGHGGSMGSMPLGCSRDDQDQETLVTHVGHYATLRRPLLDRSSLPCKGAA  638
C.salei-RDL             ........................................................................................................................  372
C.suppressalis-RDL1     ........................................................................................................................  392
C.suppressalis-RDL2     ........................................................................................................................  397
Consensus
```

```
H.sapiens-alpha1        ........................................................................................................................  416
H.sapiens-alpha2        ........................................................................................................................  414
H.sapiens-alpha3        ........................................................................................................................  451
H.sapiens-alpha4        ...........................................................................................................L....QRIKTTVN  491
H.sapiens-alpha5        ........................................................................................................................  422
H.sapiens-alpha6        ...........................................................................................................A............  387
H.sapiens-beta1         ........................................................................................................................  448
H.sapiens-beta2         ........................................................................................................................  448
H.sapiens-beta3         ........................................................................................................................  447
H.sapiens-gamma1        ...........................................................................................................RIAK.........  438
H.sapiens-gamma2        ...........................................................................................................RIAK.........  440
H.sapiens-gamma3        ...........................................................................................................DILE.........  440
R.norvegicus-alpha1     ........................................................................................................................  415
R.norvegicus-alpha2     ........................................................................................................................  467
R.norvegicus-alpha3     ........................................................................................................................  453
R.norvegicus-alpha4     ...........................................................................................................L....GRIKTTVN  489
R.norvegicus-alpha5     ........................................................................................................................  424
R.norvegicus-alpha6     ...........................................................................................................A............  387
R.norvegicus-beta1      ........................................................................................................................  448
R.norvegicus-beta2      ........................................................................................................................  448
R.norvegicus-beta3      ........................................................................................................................  447
R.norvegicus-gamma1     ...........................................................................................................RIAK.........  438
R.norvegicus-gamma2     ...........................................................................................................RIAK.........  439
R.norvegicus-gamma3     ...........................................................................................................DVSE.........  440
D.rerio-alpha1          ........................................................................................................................  418
D.rerio-alpha4          ...........................................................................................................TQRTEASSKSGAS  417
D.rerio-alpha6          ...........................................................................................................S............  331
D.rerio-beta2           ........................................................................................................................  493
D.rerio-gamma2          ...........................................................................................................RVAK.........  449
P.dominula-RDL          ........................................................................................................................  422
H.saltator-RDL          ........................................................................................................................  423
L.striatella-RDL        ........................................................................................................................  432
A.mellifera-RDL         ........................................................................................................................  421
T.castaneum-RDL         ........................................................................................................................  427
C.felis-RDL             ........................................................................................................................  428
N.lugens-RDL            ........................................................................................................................  423
D.melanogaster-RDL      ........................................................................................................................  549
B.mori-RDL2             ........................................................................................................................  440
B.mori-RDL3             ........................................................................................................................  435
B.mori-RDL1             ........................................................................................................................  427
P.xylostella-RDL2       ........................................................................................................................  465
P.xylostella-RDL1       ........................................................................................................................  430
A.funestus-RDL          ........................................................................................................................  502
D.simulans-RDL          ........................................................................................................................  549
A.aegypti-RDL           ........................................................................................................................  478
O.oryzae-RDL            ........................................................................................................................  420
M.domestica-RDL         ........................................................................................................................  518
S.furcifera-RDL         ........................................................................................................................  424
N.vitripennis-RDL       ........................................................................................................................  384
L.cuprina-RDL           ........................................................................................................................  493
S.litura-RDL1           ........................................................................................................................  430
S.litura-RDL2           ........................................................................................................................  444
T.cinnabarinus-RDL3     SGHYDRSYTCSSPPTTHRSTCTLRTCNTAHMICNHPDSIGSSGVHYPSEVRYKLSEMKTGSRGASCITTSTGEPICSGNSQGGGTGGSGGCGATSTTPLISRRSMEQS............  660
T.cinnabarinus-RDL2     ........................................................................................................................  461
T.cinnabarinus-RDL1     ........................................................................................................................  734
C.salei-RDL             ........................................................................................................................  430
C.suppressalis-RDL1     ........................................................................................................................  430
C.suppressalis-RDL2     ........................................................................................................................  441
Consensus
```

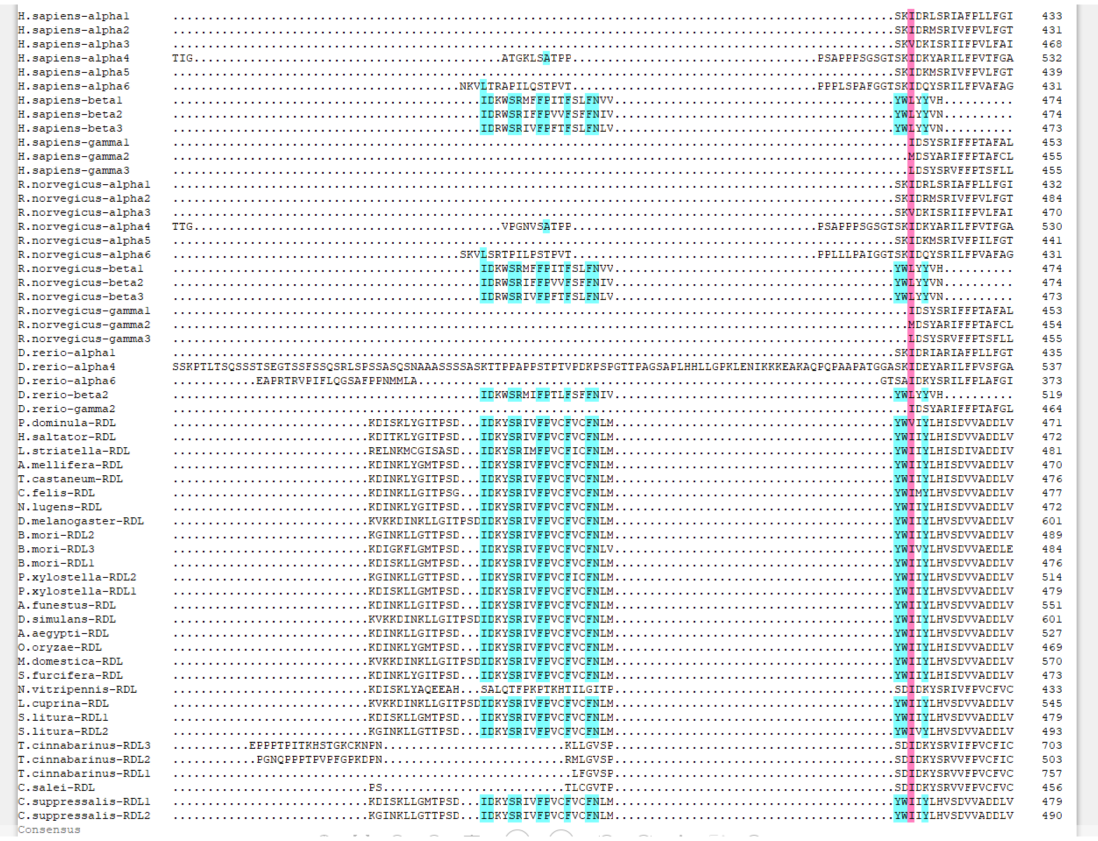

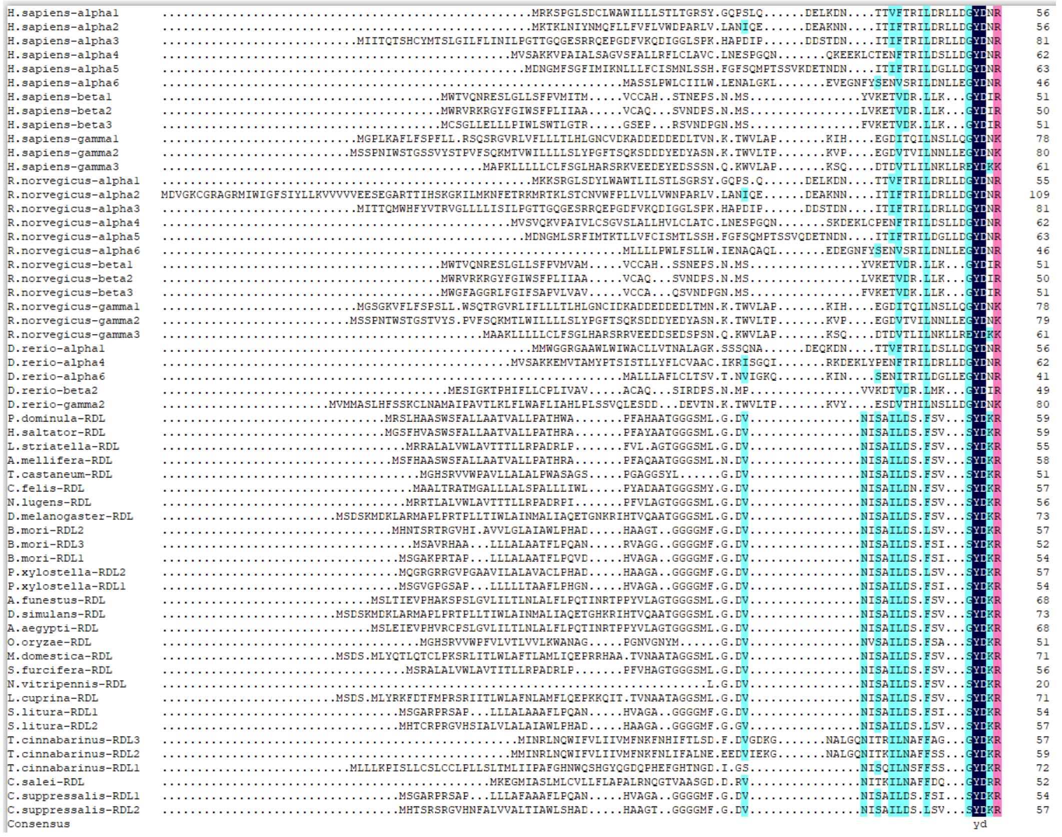

H.sapiens-alpha1	FNLVYWATYLNREPQLKAPTPHQ...	456
H.sapiens-alpha2	FNLVYWATYLNREPVLGVSP......	451
H.sapiens-alpha3	FNLVYWATYVNRESAIKGMIRKQ...	491
H.sapiens-alpha4	FNMVYWVVYLSKDTMEKSESLM....	554
H.sapiens-alpha5	FNLVYWATYLNREPVIKGAASPK...	462
H.sapiens-alpha6	FNLVYWVVYLSKDTMEVSSSVE....	453
H.sapiens-beta1	....	474
H.sapiens-beta2	....	474
H.sapiens-beta3	....	473
H.sapiens-gamma1	FNLVYWVGYLYL..............	465
H.sapiens-gamma2	FNLVYWVSYLYL..............	467
H.sapiens-gamma3	FNLVYWVGYLYL..............	467
R.norvegicus-alpha1	FNLVYWATYLNREPQLKAPTPHQ...	455
R.norvegicus-alpha2	FNLVYWATYLNREPVLGVSP......	504
R.norvegicus-alpha3	FNLVYWATYVNRESAIKGMIRKQ...	493
R.norvegicus-alpha4	FNMVYWVVYLSKDTMEKSESLM....	552
R.norvegicus-alpha5	FNLVYWATYLNREPVIKGATSPK...	464
R.norvegicus-alpha6	FNLVYWIVYLSKDTMEVSSTVE....	453
R.norvegicus-beta1	....	474
R.norvegicus-beta2	....	474
R.norvegicus-beta3	....	473
R.norvegicus-gamma1	FNLVYWVGYLYL..............	465
R.norvegicus-gamma2	FNLVYWVSYLYL..............	466
R.norvegicus-gamma3	FNLVYWVGYLYL..............	467
D.rerio-alpha1	FNLVYWATYLNKKPKLQGMNLQPH..	459
D.rerio-alpha4	FNMVYWVVYLSKDTMEAKGA......	557
D.rerio-alpha6	FNLVYWYIYLSKDTLEKLRDIE....	395
D.rerio-beta2	....	519
D.rerio-gamma2	FNVVYWFSYLYL..............	476
P.dominula-RDL	LLKEAK....................	477
H.saltator-RDL	LLEEAK....................	478
L.striatella-RDL	MLEPDK....................	487
A.mellifera-RDL	LLEEAK....................	476
T.castaneum-RDL	LLEEDK....................	482
C.felis-RDL	LLGEDK....................	483
N.lugens-RDL	LLEEDK....................	478
D.melanogaster-RDL	LLGEE.....................	606
B.mori-RDL2	LLGEEN....................	495
B.mori-RDL3	LLEDK.....................	489
B.mori-RDL1	LLGEDK....................	482
P.xylostella-RDL2	LLGDIN....................	520
P.xylostella-RDL1	LLEEDK....................	485
A.funestus-RDL	LLGEEK....................	557
D.simulans-RDL	LLGEE.....................	606
A.aegypti-RDL	LLGEEK....................	533
O.oryzae-RDL	LLEVDK....................	475
M.domestica-RDL	LLGEEK....................	576
S.furcifera-RDL	LLEEDK....................	479
N.vitripennis-RDL	FNLMYWIIYLHISDVVADDLVLLEEA	459
L.cuprina-RDL	LLGEE.....................	550
S.litura-RDL1	LLEEDK....................	485
S.litura-RDL2	LLGEEN....................	499
T.cinnabarinus-RDL3	FNLMYWIIYLHISNEPNPDLIQLGS.	728
T.cinnabarinus-RDL2	FNLMYWIIYMHISASSSPN.......	522
T.cinnabarinus-RDL1	FNLMYWIIFLHISSILEPGADEES..	781
C.salei-RDL	FNLMYWIIYLHISDV....LVEVQEN	478
C.suppressalis-RDL1	LLEEDK....................	485
C.suppressalis-RDL2	LLGEEN....................	496
Consensus		
