# Supplementary material for: G3’MTMD3 in the insect GABA receptor subunit, RDL, confers resistance to broflanilide and fluralaner
Source: PLoS Genet. 2023 Jun 29;19(6):e1010814. doi: 10.1371/journal.pgen.1010814 (PMC10337980; doi:10.1371/journal.pgen.1010814)
Supplement: S1 Table — (PDF) [file pgen.1010814.s009.pdf]

**S1 Table. Prediction of potential amino acid residues in *Cs*RDL interacting with fluralaner**

| Transmembrane domain | Position on *Cs*RDL | WT | Mutation | Binding energy | | | | |
|---|---|---|---|---|---|---|---|---|
| | | | | WT | Mutation | | | **No.** |
| TMD1 | 258 (228) | I | T | 4.5215(I) | 4.5902(T) | | | **1** |
| | 260 (230) | I | L/M | 4.2084(I) | 5.3753(L) | 4.6323(M) | | **2** |
| | 262 (232) | S | C | 4.4730(S) | 4.4480(C) | | | **3** |
| | 263 (233) | G | I | 4.7443(G) | 5.4000(I) | | | **4** |
| | 264 (234) | L | M | 3.9282(L) | 3.6924(M) | | | **5** |
| | 265 (235) | I | T | 3.7468(I) | 4.8598(M) | | | **6** |
| | 268 (238) | I | L | 4.8808(I) | 4.2732(L) | | | **7** |
| | 275 (245) | L | I | 1.4511(L) | 3.6435(I) | | | **8** |
| TMD2 | 284 (254) | V | T | 3.5264(V) | 4.1300(T) | | | **9** |
| | 288 (258) | V | I | 5.4886(V) | 2.9646(I) | | | **10** |
| | 298 (268) | M | S/N | 5.4843(M) | 4.6268(S) | 4.4541(N) | | **11** |
| | 299 (269) | S | I/T | 6.1474(S) | 5.4397(I) | 5.7228(T) | | **12** |
| | 301 (271) | T | A/L | 6.6488(T) | 6.2552(A) | 5.8912(L) | | **13** |
| | 302 (272) | N | R | 3.9210(N) | 5.0899(R) | | | **14** |
| | 303 (273) | A | N/E/K | 4.9935(A) | 3.7599(N) | 4.1328(E) | 4.5773(K) | **15** |
| | 304 (274) | A | S/T | 4.8820(A) | 3.7685(S) | 4.8886(T) | | **16** |
| TMD3 | 317 (287) | Y | F | 4.8300(Y) | 7.2348(F) | | | **17** |
| | 318 (288) | L | I/V | 3.7757(L) | 5.4566(I) | 4.8602(V) | | **18** |
| | 319 (289) | G | A/M/S | 5.0231(G) | 4.8663(A) | 5.6859(M) | 7.5320(S) | **19** |
| | 320 (290) | T | V/G | 4.4847(T) | 4.9667(V) | 4.6417(G) | | **20** |
| | 323 (293) | V | A | 4.2857(V) | 3.8009(A) | | | **21** |
| | 324 (294) | M | F | 4.4061(M) | 5.2403(F) | | | **22** |
| | 327 (297) | A | S | 3.5754(A) | 2.5077(S) | | | **23** |
| | 328 (298) | S | A | 1.9168(S) | 2.0141(A) | | | **24** |
| | 330 (300) | L | I/M | 4.1681(L) | 3.5937(I) | 3.9840(M) | | **25** |
| | 336 (306) | G | N | 4.3093(G) | 5.1995(N) | | | **26** |
| | 338 (308) | M | I | 4.4562(M) | 5.0653(I) | | | **27** |
| | 339 (309) | A | F | 5.7805(A) | 5.8155(F) | | | **28** |
| TMD4 | 462 (325) | V | F | 4.7601(V) | 4.6287(F) | | | **29** |
| | 466 (329) | C | T | 3.0400(C) | 4.7353(T) | | | **30** |
| | 468 (331) | V | S | 3.8259(V) | 3.4912(S) | | | **31** |
| | 469 (332) | C | F/L | 3.6769(C) | 4.1046(F) | 5.1194(L) | | **32** |
| | 473 (336) | M | V | 3.3124(M) | 6.2653(V) | | | **33** |
| | 474 (337) | Y | S | 4.8024(Y) | 5.5412(S) | | | **34** |
| | 477 (340) | I | D/Y | 6.0092(I) | 2.9393(D) | 4.8363(Y) | | **35** |

Note, amino acid residues in red were selected for functional expression studies.
