# Supplementary material for: G3’MTMD3 in the insect GABA receptor subunit, RDL, confers resistance to broflanilide and fluralaner
Source: PLoS Genet. 2023 Jun 29;19(6):e1010814. doi: 10.1371/journal.pgen.1010814 (PMC10337980; doi:10.1371/journal.pgen.1010814)
Supplement: S2 Table — (PDF) [file pgen.1010814.s010.pdf]

S2 Table. Potencies of GABA on *X. laevis* oocytes injected with *Cs*RDL cRNA

	cRNA	EC_{50} (95% CI) (μM)	Hill Slope (95% CI)	I_{max} ± SE (nA)	Number
**1**	wild-type	18.07 (13.94-23.42)	1.86 (1.09-2.62)	-5663 ± 394	6
**2**	I258T	26.69 (17.96-39.67)	0.95 (0.63-1.28)	$-575 \pm 223^{****}$	5
**3**	L275I	$50.46^{\#}$ (43.73-58.23)	2.48 (1.89-3.06)	-4526 ± 791	5
**4**	V288I	$170.80^{\#}$ (153.80-189.70)	1.96 (1.63-2.30)	$-2134 \pm 775^{**}$	5
**5**	M298N	$161.60^{\#}$ (117.50-222.40)	1.15 (0.77-1.53)	$-186 \pm 90^{****}$	5
**6**	AA303-304NS	$319.80^{\#}$ (268.60-380.70)	1.76 (1.26-2.26)	$-2698 \pm 961^{**}$	5
**7**	G3'M_{TMD3}	$615.90^{\#}$ (506.80-748.50)	1.41 (1.08-1.74)	-6265 ± 753	5
**8**	G319S	$1156.00^{\#}$ (977.00-1368.00)	2.84 (1.19-4.48)	$-3413 \pm 1025^{*}$	5
**9**	A327S	$108.60^{\#}$ (89.24-132.20)	2.40 (1.10-3.70)	$-1735 \pm 547^{****}$	5
**10**	G336N	$140.00^{\#}$ (124.10-158.10)	2.45 (1.85-3.05)	-4949 ± 1366	5
**11**	MA338-339IF	$121.40^{\#}$ (90.61-162.70)	2.02 (0.89-3.15)	$-2021 \pm 739^{***}$	5
**12**	M473V	17.58 (16.07-19.24)	2.03 (1.72-2.33)	$-2363 \pm 1101^{*}$	5
**13**	I477D	ND	ND	ND	12

CI, confidence interval; ND, not detectable.

# indicates significant difference relative to wild-type *Cs*RDL as determined by the 95% CI without overlapping.

*, **, *** and **** indicate significant difference relative to wild-type *Cs*RDL as determined by Student's *t*-test with SPSS 17.0 (SPSS Inc., Chicago, IL) when $P < 0.05$, $P < 0.01$, $P < 0.001$ or $P < 0.0001$, respectively.
