# Supplementary material for: G3’MTMD3 in the insect GABA receptor subunit, RDL, confers resistance to broflanilide and fluralaner
Source: PLoS Genet. 2023 Jun 29;19(6):e1010814. doi: 10.1371/journal.pgen.1010814 (PMC10337980; doi:10.1371/journal.pgen.1010814)
Supplement: S3 Table — (PDF) [file pgen.1010814.s011.pdf]

S3 Table. Inhibition of GABA-induced currents by fluralaner in *X. laevis* oocytes injected with *Cs*RDL cRNA

	cRNA	IC_{50} (95% CI) (nM)	Number		cRNA	IC_{50} (95% CI) (nM)	Number
**1**	wild-type	4.20 (2.56-6.88)	6	**7**	G3'M_{TMD3}	>10,000*	5
**2**	I258T	9.39 (4.47-19.72)	4	**8**	G319S	1.37 (0.73-2.57)	5
**3**	L275I	14.54 (4.78-44.26)	4	**9**	A327S	4.92 (2.93-8.24)	4
**4**	V288I	13.08 (5.07-33.70)	4	**10**	G336N	5.74 (3.41-9.68)	5
**5**	M298N	2.06 (0.97-4.38)	3	**11**	M473V	13.20 (6.24-27.92)	5
**6**	AA303-304NS	24.84* (14.07-43.86)	4	**12**	MA338-339IF	1.85 (0.73-4.67)	4

CI, confidence interval

* indicates significant difference relative to wild-type *Cs*RDL as determined by the 95% CI without overlapping.
