# Supplementary material for: G3’MTMD3 in the insect GABA receptor subunit, RDL, confers resistance to broflanilide and fluralaner
Source: PLoS Genet. 2023 Jun 29;19(6):e1010814. doi: 10.1371/journal.pgen.1010814 (PMC10337980; doi:10.1371/journal.pgen.1010814)
Supplement: S4 Table — (PDF) [file pgen.1010814.s012.pdf]

S4 Table. Inhibition of GABA-induced currents by avermectin and fipronil in *X. laevis* oocytes injected with G3'M$_{TMD3}$ *Cs*RDL cRNAs.

cRNA	IC_{50} (95% CI) (nM) (number of oocytes)	
	**Fipronil**	**Avermectin**
wild-type	10.02 (7.17-14.00) (5)	69.90 (46.67-104.70) (4)
G3'M$_{TMD3}$	22.42* (14.15-35.51) (6)	> 10,000* (5)

CI, confidence interval.

* indicates significant difference relative to wild-type *Cs*RDL as determined by the 95% CI without overlapping.
