# Supplementary material for: G3’MTMD3 in the insect GABA receptor subunit, RDL, confers resistance to broflanilide and fluralaner
Source: PLoS Genet. 2023 Jun 29;19(6):e1010814. doi: 10.1371/journal.pgen.1010814 (PMC10337980; doi:10.1371/journal.pgen.1010814)
Supplement: S5 Table — (PDF) [file pgen.1010814.s013.pdf]

S5 Table. Potencies of GABA and fluralaner on wild-type or G3'M$_{TMD3}$ RDL cRNA from different species expressed in *X. laevis* oocytes.

cRNA	Fluralaner	GABA	
	IC_{50} (95% CI) (nM)	EC_{50} (95% CI) (μM)	Hill Slope (95% CI)
wild-type *Cs*RDL	4.51 (3.00-6.79)	38.32 (34.43-42.66)	1.87 (1.55-2.20)
G3'M$_{TMD3}$ *Cs*RDL	> 10,000*	1306* (1208-1413)	2.40 (1.99-2.81)
wild-type *Ls*RDL	3.27 (1.90-5.62)	31.95 (27.90-36.60)	1.97 (1.45-2.48)
G3'M$_{TMD3}$ *Ls*RDL	> 10,000*	756.60* (687.20-833.10)	2.30 (1.85-2.75)
wild-type *Am*RDL	9.38 (6.68-13.17)	13.37 (12.31-14.53)	2.62 (2.13-3.12)
G3'M$_{TMD3}$ *Am*RDL	> 10,000*	267.50* (203.90-351.00)	1.98 (0.939-3.02)
wild-type *Dm*RDL	6.96 (2.70-17.95)	23.51 (20.88-26.48)	1.95 (1.53-2.36)
G3'M$_{TMD3}$ *Dm*RDL	> 10,000*	234.40* (198.00-277.40)	2.33 (1.48-3.18)
wild-type *Tu*RDL	2.53 (1.53-4.19)	295.10 (232.70-374.10)	1.51 (1.02-2.00)
G3'M$_{TMD3}$ *Tu*RDL	> 10,000*	2360* (2094-2661)	2.23 (1.67-2.78)

Note, The different species used were *Chilo suppressalis* (*Cs*), *Laodelphax striatellus* (*Ls*), *Apis mellifera* (*Am*), *Drosophila melanogaster* (*Dm*) and *Tetranychus urticae* (*Tu*).

CI, confidence interval.

* indicates significant difference relative to wild-type RDL as determined by the 95% CI without overlapping.
