# Supplementary material for: G3’MTMD3 in the insect GABA receptor subunit, RDL, confers resistance to broflanilide and fluralaner
Source: PLoS Genet. 2023 Jun 29;19(6):e1010814. doi: 10.1371/journal.pgen.1010814 (PMC10337980; doi:10.1371/journal.pgen.1010814)
Supplement: S6 Table — (PDF) [file pgen.1010814.s014.pdf]

S6 Table. Potencies of GABA on heteromeric *Mm*α1β2 and *Mm*α1β2-M3'G_{TMD3} receptors expressed in *X. laevis* oocytes.

cRNA	EC_{50} (95% CI) (μM)	Hill Slope (95% CI)	Number
*Mm*α1β2	9.18 (7.76-10.85)	1.44 (1.13-1.75)	7
*Mm*α1β2-M3'G_{TMD3}	3.25* (2.58-4.09)	0.97 (0.77-1.17)	7

CI, confidence interval.

* indicates significant difference relative to *Mm*α1β2 as determined by the 95% CI without overlapping.
