# Supplementary material for: G3’MTMD3 in the insect GABA receptor subunit, RDL, confers resistance to broflanilide and fluralaner
Source: PLoS Genet. 2023 Jun 29;19(6):e1010814. doi: 10.1371/journal.pgen.1010814 (PMC10337980; doi:10.1371/journal.pgen.1010814)
Supplement: S7 Table — (PDF) [file pgen.1010814.s015.pdf]

S7 Table. Inhibition by fluralaner on GABA-induced currents in *X. laevis* oocytes injected with *Mm*α1β2 or *Mm*α1β2-M3'G_{TMD3}

	Mean ± SE (%) (number)			
**cRNA**	**10^{-6} M**	**10^{-7} M**	**10^{-8} M**	**10^{-9} M**
*Mm*α1β2	27.17 ± 4.42 (12)	26.22 ± 3.05 (15)	11.97 ± 2.00 (12)	3.23 ± 1.46 (5)
*Mm*α1β2-M3'G_{TMD3}	40.75 ± 4.50 (11)	28.69 ± 4.71 (10)	26.68 ± 3.90** (10)	22.59 ± 3.25** (8)

** indicates significant difference relative to *Mm*α1β2 at the same concentration as determined by Student's *t*-test with SPSS 17.0 (SPSS Inc., Chicago, IL) when $P < 0.01$.
