# Supplementary material for: G3’MTMD3 in the insect GABA receptor subunit, RDL, confers resistance to broflanilide and fluralaner
Source: PLoS Genet. 2023 Jun 29;19(6):e1010814. doi: 10.1371/journal.pgen.1010814 (PMC10337980; doi:10.1371/journal.pgen.1010814)
Supplement: S8 Table — (PDF) [file pgen.1010814.s016.pdf]

S8 Table. Toxicity of four insecticides to w^{1118} adults and heterozygous adults bearing G3'$_{TMD3}$ mutations

Insecticide	Genotype	Slope ± SE	LC_{50} (95% CI[†]) (mg/L)	χ^2 (df)	*P* value	RR[‡]
Fipronil	w^{1118}	5.77±0.71	0.82 (0.76-0.89)	7.73 (13)	0.861	1.00
	G3'M$_{TMD3}$/TM2 Ubx^{130}	5.23±0.67	0.78 (0.72-0.85)	5.69 (13)	0.957	0.95
	G3'Q$_{TMD3}$/TM2 Ubx^{130}	4.61±0.63	0.84 (0.76-0.92)	5.48 (13)	0.963	1.02
	G3'S$_{TMD3}$/TM2 Ubx^{130}	5.38±0.68	0.80 (0.74-0.87)	2.93 (13)	0.998	0.98
Avermectin	w^{1118}	1.74±0.23	33.80 (26.36-45.33)	7.22 (13)	0.891	1.00
	G3'M$_{TMD3}$/TM2 Ubx^{130}	1.65±0.22	30.64 (23.70-41.14)	6.08 (13)	0.943	0.91
	G3'Q$_{TMD3}$/TM2 Ubx^{130}	1.70±0.23	35.00 (27.16-47.36)	7.42 (13)	0.879	1.04
	G3'S$_{TMD3}$/TM2 Ubx^{130}	1.60±0.22	29.67 (22.81-39.90)	8.14 (13)	0.834	0.88
Fluralaner	w^{1118}	2.07±0.25	1.89 (1.50-2.33)	6.87 (13)	0.909	1.00
	G3'M$_{TMD3}$/TM2 Ubx^{130}	2.21±0.27	1.64 (1.31-2.00)	4.21 (13)	0.989	0.87
	G3'Q$_{TMD3}$/TM2 Ubx^{130}	2.27±0.27	1.76 (1.42-2.14)	9.19 (13)	0.759	0.93
	G3'S$_{TMD3}$/TM2 Ubx^{130}	2.26±0.27	1.75 (1.41-2.13)	9.42 (13)	0.741	0.93
Broflanilide	w^{1118}	2.40±0.27	0.81 (0.66-0.97)	8.86 (13)	0.783	1.00
	G3'M$_{TMD3}$/TM2 Ubx^{130}	2.43±0.28	0.75 (0.61-0.90)	4.90 (13)	0.977	0.93
	G3'Q$_{TMD3}$/TM2 Ubx^{130}	2.44±0.28	0.81 (0.67-0.98)	7.58 (13)	0.870	1.00

	G3'S$_{TMD3}$/TM2 *Ubx*^{130}	2.57±0.29	0.86 (0.72-1.03)	7.73(13)	0.861	1.06

† CI, confidence intervals.
‡ RR indicates resistance ratio: resistance ratio = LC_{50} value of a heterozygous G3'$_{TMD3}$ mutant/LC_{50} value of the wild-type *w*^{1118}.

.
