# Supplementary material for: G3’MTMD3 in the insect GABA receptor subunit, RDL, confers resistance to broflanilide and fluralaner
Source: PLoS Genet. 2023 Jun 29;19(6):e1010814. doi: 10.1371/journal.pgen.1010814 (PMC10337980; doi:10.1371/journal.pgen.1010814)
Supplement: S9 Table — (PDF) [file pgen.1010814.s017.pdf]

**S9 Table. Survival rate of larvae of $w^{1118}$ and homozygous for G3'$_{TMD3}$ mutations.**

| Genotype | Survival number per day after hatching (n = 20 for each replicate) | | | | | | | |
|---|---|---|---|---|---|---|---|---|
| | **0 d** | **1 d** | **2 d** | **3 d** | **4 d** | **5 d** | **6 d** | **7 d** |
| $w^{1118}$-replicate 1 | 20 | 20 | 19 | 19 | 19 | 19 | 19 | 19 |
| $w^{1118}$-replicate 2 | 20 | 20 | 20 | 20 | 20 | 20 | 20 | 20 |
| $w^{1118}$-replicate 3 | 20 | 20 | 19 | 19 | 19 | 19 | 19 | 19 |
| G3'M$_{TMD3}$-replicate 1 | 20 | 19 | 16 | 16 | 3 | 1 | 0 | 0 |
| G3'M$_{TMD3}$-replicate 2 | 20 | 18 | 17 | 15 | 3 | 2 | 1 | 0 |
| G3'M$_{TMD3}$-replicate 3 | 20 | 18 | 15 | 13 | 2 | 2 | 0 | 0 |
| G3'Q$_{TMD3}$-replicate 1 | 20 | 17 | 10 | 5 | 4 | 3 | 2 | 0 |
| G3'Q$_{TMD3}$-replicate 2 | 20 | 15 | 10 | 6 | 4 | 3 | 0 | 0 |
| G3'Q$_{TMD3}$-replicate 3 | 20 | 15 | 9 | 7 | 6 | 4 | 1 | 0 |
| G3'S$_{TMD3}$-replicate 1 | 20 | 8 | 6 | 3 | 2 | 1 | 0 | 0 |
| G3'S$_{TMD3}$-replicate 2 | 20 | 10 | 7 | 5 | 3 | 2 | 0 | 0 |
| G3'S$_{TMD3}$-replicate 3 | 20 | 11 | 7 | 4 | 2 | 0 | 0 | 0 |
