# Supplementary material for: G3’MTMD3 in the insect GABA receptor subunit, RDL, confers resistance to broflanilide and fluralaner
Source: PLoS Genet. 2023 Jun 29;19(6):e1010814. doi: 10.1371/journal.pgen.1010814 (PMC10337980; doi:10.1371/journal.pgen.1010814)
Supplement: S10 Table — (PDF) [file pgen.1010814.s018.pdf]

S10 Table. Crawling speed of larvae of w^{1118} and homozygous for $G3'_{TMD3}$ mutations

Genotype	Crawling speed (± SE) (mm/min)	Number
w^{1118}	2.92 ± 0.21	20
$G3'M_{TMD3}$	0.52 ± 0.046 ***	20
$G3'Q_{TMD3}$	0.85 ± 0.096 ***	20
$G3'S_{TMD3}$	1.13 ± 0.12 ***	20

*** indicates significant difference relative to w^{1118} as determined by Student's *t*-test with SPSS 17.0 (SPSS Inc., Chicago, IL) when $P < 0.001$.
