# Supplementary material for: G3’MTMD3 in the insect GABA receptor subunit, RDL, confers resistance to broflanilide and fluralaner
Source: PLoS Genet. 2023 Jun 29;19(6):e1010814. doi: 10.1371/journal.pgen.1010814 (PMC10337980; doi:10.1371/journal.pgen.1010814)
Supplement: S11 Table — (PDF) [file pgen.1010814.s019.pdf]

S11 Table. Primers used in this study

Name	Forward primer (5'-3')	Reverse primer (5'-3')	Function
p-I258T	ATTACTTGATTCAAACTTATATTCCA	GTTTGAATCAAGTAATATCCCATAGA	*Cs*RDL-I258T
p-L275I	TGGGTGTCTTTTTGGATCAATAGAAATG	GATCCAAAAAGACACCCACGATATGATG	*Cs*RDL-L275I
p-V288I	AGAGTGTCGTTGGGCATAACAACTGT	TGCCCAACGACACTCTAGCTGGTGTC	*Cs*RDL-V288I
p-M298N	CTATGACCACCCTAAACTCTTCAACAA	GTTTAGGGTGGTCATAGTCAAAACAGT	*Cs*RDL-M298N
p-AA303-304NS	ATGTCTTCAACAAATAATTCTTTGCCCAA	AATTATTTGTTGAAGACATTAGGGTGGTC	*Cs*RDL-AA303-304NS
p-G319M	ATCGATGTCTACCTAATGACCTGCTTCG	CATTAGGTAGACATCGATAGATTTCACA	*Cs*RDL-G319M
p-G319S	ATCGATGTCTACCTATCAACCTGCTTCG	TGATAGGTAGACATCGATAGATTTCACA	*Cs*RDL-G319S
p-A327S	TTCGTGATGGTCTTCTCAAGCCTATT	AGAAGACCATCACGAAGCAGGTTCCT	*Cs*RDL-A327S
p-G336N	GAATATGCTACTGTTAACTATATGGCT	TTAACAGTAGCATATTCTAATAGGCTT	*Cs*RDL-G336N
p-MA338-339IF	TACTGTTGGCTATATATTTAAACGAATA	AATATATAGCCAACAGTAGCATATTCTA	*Cs*RDL-MA338-339IF
p-M473V	GTCTGCTTTAATCTTGTGTATTGGAT	CAAGATTAAAGCAGACGAAGCAAACG	*Cs*RDL-M473V
p-I477D	CTTATGTATTGGATAGATTATCTTCAC	TCTATCCAATACATAAGATTAAAGCAG	*Cs*RDL-I477D
*Tu*RDL	TTGGCTGTGCTTTTCTCCTT	GCATTTTCCAGCTTCTGCTT	*Tu*RDL
pGH19-*Tu*RDL	*ATTCCCCGGGGATCC*GAATTCGCCACCATGATG ATGTTGATGCTGAT	*TCGGCGATCGGGCCC*TCTAGATTAAGACTCTT CGTCGGCC	pGH19-*Tu*RDL
p-*Tu*RDL-G339M	ATCGATGTTTTCCTGATGACTTGTTTCG	CATCAGGAAAACATCGATACTCTTTACG	*Tu*RDL-G339M
*Dm*RDL	ACCACCATGAGTGATTCAAAAATGG	AGTGCCCTGCCAAGTTTGACTCT	*Dm*RDL
pGH19-*Dm*RDL	*ATTCCCCGGGGATCC*GAATTCGCCACCATGAGT GATTCAAAAATGG	*TCGGCGATCGGGCCC*TCTAGACTACTCCTCGC CCAGAAGCA	pGH19-*Dm*RDL
p-*Dm*RDL-G335M	ATTGACGTCTATCTGATGACATGCTTCG	CATCAGATAGACGTCAATCGATTTGACG	*Dm*RDL-G335M
p-*Am*RDL-G320M	ATCGACGTTTACCTGATGACATGTTTCG	CATCAGGTAAACGTCGATCGACTTGACG	*Am*RDL-G320M
p-*Ls*RDL-G317M(9c)	ATCGACGTCTACCTGATGACCTGTTTCG	CATCAGGTAGACGTCGATCGACTTGACG	*Ls*RDL-G317M(9c)
p-*Cs*RDL1-G316M	ATCGACGTGTATTTGATGACCTGTTTCG	CATCAAATACACGTCGATAGATTTCACG	*Cs*RDL1-G316M
*Mm*α1	ATGAAGAAAAGTCGGGGTCTC	CTATTGATGGGGTGTGGGGGCTT	*Mm*α1
pGH19-*Mm*α1	*ATTCCCCGGGGATCC*GAATTCATGAAGAAAAG TCGGGGTCTC	*TCGGCGATCGGGCCC*TCTAGACTATTGATGGG GTGTGGGGGCTT	pGH19-*Mm*α1

*Mm*β2	ATGTGGAGAGTCCGGAAAAGG	TTAGTTCACATAGTAAAGCCAA	*Mm*β2
pGH19-*Mm*β2	*ATTCCCCGGGGATCC*GAATTCATGTGGAGAGTCCGGAAAAGG	*TCGGCGATCGGGCCC*TCTAGATTAGTTCACATAGTAAAGCCAA	pGH19-*Mm*β2
p-*Mm*β2-M310G	ATTGACATGTACCTAGGTGGGTGCTTTG	ACCTAGGTACATGTCAATGGCTTTGACA	*Mm*β2-M310G
Dmrdl-gRNA1	TAATACGACTCACTATAAAATCGATTGACGTCTATCTGTTTTAGAGCTAGAAATAGCAAGTTAAAATAA	AGCACCGACTCGGTGCCACTTTTTCAAGTTGATAACGGACTAGCCTTATTTTAACTTGCTATTTCTAGCTCTAA	Generation of templates for gRNA *in vitro* transcription
Dmrdl-gRNA2	TAATACGACTCACTATATCTGGGAACATGCTTCGTTAGTTTTAGAGCTAGAAATAGCAAGTTAAAATAA		
Dmrdl-G3-5F	CCTCTTCGCTATTACGCCAGCAACGATTATTGCTTCGGTTAAGTC	AAACAAGTCATTAAGTACACATCTATCGATTTGACGTACGAAATC<br>AAACAAGTCGATAAGTACACATCTATCGATTTGACGTACGAAATC	Generation of DNA fragments for the assembly of donor plasmids
Dmrdl-G3M-3F	GTGTACTTAATGACTTGTTTTGTCATGGTCTTTGCCAGTCTACTG	GCTATGACCATGATTACGCCATTCGATTCTAGGTGCGAGGTG	
Dmrdl-G3S-3F	GTGTACTTATCGACTTGTTTTGTCATGGTCTTTGCCAGTCTACTG		
pBSK-s	TGGCGTAATCATGGTCATAGC	CTGGCGTAATAGCGAAGAGG	
pCFD4-gRNAs	GATATCCGGGTGAACTTCGAAATCGATTGACGTCTATCTGTTTTAGAGCTAGAAATAGCAAG	CTATTTCTAGCTCTAAAACTAACGAAGCATGTTCCCAGACGACGTTAAATTGAAAATAGGTC	Generation of DNA fragment with gRNA sequences fused
G3QDonor	ATAGATGTGTACTTACAGACTTGTTTT	TGTAAGTACACATCTATCGATTTGACG	Generation of mutated donor plasmid
rdl335det	GCAACTATTCGCGTTTAGCC	CTGGCTGTTGATCGACGACT	Amplification of the genomic region of *Drosophila* for sequencing

Note, the restriction enzyme sites for *Eco* RI and *Xba* I are shown in red, and the Kozak sequence is indicated in green, the nucleotide sequences matching the pGH19 vector are in italics. The blue nucleotides are the mutant sites.
