# Supplementary material for: G3’MTMD3 in the insect GABA receptor subunit, RDL, confers resistance to broflanilide and fluralaner
Source: PLoS Genet. 2023 Jun 29;19(6):e1010814. doi: 10.1371/journal.pgen.1010814 (PMC10337980; doi:10.1371/journal.pgen.1010814)
Supplement: S1 Note — Note, the amino acids marked as yellow are the TMDs, and all the potential amino acid residues predicted to interact with fluralaner are shown in red, while the G3’TMD3 (wild-type) residue is marked in green and the M3’TMD3 (mutant) residue is marked in blue. (PDF) [file pgen.1010814.s020.pdf]

S1 Note

>*Chilo suppressalis* RDL ***Chilo suppressali* RDL-wild-type**

MHTSRSRGVHNFALVVALTIAWLSHADHAAGTGGGGMFGDVNISAILDSLSVSYDKRVRPNY
GGPPVDVGVTMYVLSISSLSEVKMDFTLDFYFRQFWTDPRLAYKKRTGVETLSVGSEFIRNIW
VPDTFFVNEKQSYFHIATTSNEFIRIHHSGSITRSIRLTITASCPMDLQYFPMDRQLCNIEIESFGYT
MRDIRYKWNEGPNSVGVSSEVSLPQFKVLGHRQRAMEISLTTGNYSRLACEIQFVRSMGYYLI
QIYIPSGLIVIISWVSFWLNRNATPARVSLGVTTVLTMTTLMSSTNAALPKISYVKSIDVYLGTCF
VMVFASLLEYATVGYMAKRIQMRKQRFTAVQKMAAEKKMHIDGPPGTSEPLPPPRTSTLNRPL
PPSRSSEVRFKVHDPKAYSKGGTLENTINGARAPPPPPVPQPEEDPAPPPHLLQASKGINKLLGT
TPSDIDKYSRIVFPVCFVCFNLMYWIIYLHVSDVVADDLVLLGEEN

***Chilo suppressalis* RDL-G3'M$_{TMD3}$**

MHTSRSRGVHNFALVVALTIAWLSHADHAAGTGGGGMFGDVNISAILDSLSVSYDKRVRPNY
GGPPVDVGVTMYVLSISSLSEVKMDFTLDFYFRQFWTDPRLAYKKRTGVETLSVGSEFIRNIW
VPDTFFVNEKQSYFHIATTSNEFIRIHHSGSITRSIRLTITASCPMDLQYFPMDRQLCNIEIESFGY
TMRDIRYKWNEGPNSVGVSSEVSLPQFKVLGHRQRAMEISLTTGNYSRLACEIQFVRSMGYYL
IQIYIPSGLIVIISWVSFWLNRNATPARVSLGVTTVLTMTTLMSSTNAALPKISYVKSIDVYLMT
CFVMVFASLLEYATVGYMAKRIQMRKQRFTAVQKMAAEKKMHIDGPPGTSEPLPPPRTSTLN
RPLPPSRSSEVRFKVHDPKAYSKGGTLENTINGARAPPPPPVPQPEEDPAPPPHLLQASKGINKL
LGTTPSDIDKYSRIVFPVCFVCFNLMYWIIYLHVSDVVADDLVLLGEEN

***Laodelphax striatellus* RDL-wild-type**

MRRALALVWLAVTTTLLRPADRLPFVLAGTGGGSMLGDVNISAILDSFSVSYDKRVRPNYGGP
PVEVGVTMYVLSISSLSEVKMDFTLDFYFRQFWTDPRLAFRKRPGVETLSVGSEFIKNIWVPDT
FFVNEKQSYFHIATTSNEFIRIHHSGSITRSIRLTITASCPMNLQYFPMDRQLCHIEIESFGYTMRDI
RYKWNEGPNSVGVSNEVSLPQFKVLGHRQRAMEISLTTGNYSRLACEIQFVRSMGYYLIQIYIP
SGLIVIISWVSFWLNRNATPARVALGVTTVLTMTTLMSSTNAALPKISYVKSIDVYLGTCFVMV
FASLLEYATVGYMAKRIQMRKNRFMAIQKIAEQKQKQGMEAHAGHPPGVPGGGDPADHAPK
QTATRYKTLDSKGHYKSGTLDSRTYGRPDKEAPAPPPPPPPPELNRSERELNKMC

GISASDIDKYSRIMFPVCFICFNLMYWIIYLHISDIVADDIVMLEPDK

***Laodelphax striatellus* RDL-G3’M$_{TMD3}$**

MRRALALVWLAVTTTLLRPADRLPFVLAGTGGGSMLGDVNISAILDSFSVSYDKRVRPNYGGPPVEVGVTMYVLSISSLSEVKMDFTLDFYFRQFWTDPRLAFRKRPGVETLSVGSEFIKNIWVPDTFFVNEKQSYFHIATTSNEFIRIHHSGSITRSIRLTITASCPMNLQYFPMDRQLCHIEIESFGYTMRDIRYKWNEGPNSVGVSNEVSLPQFKVLGHRQRAMEISLTTGNYSRLACEIQFVRSMGYYLIQIYIPSGLIVIISWVSFWLNRNATPARVALGVTTVLTMTTLMSSTNAALPKISYVKSIDVYLMTCFVMVFASLLEYATVGYMAKRIQMRKNRFMAIQKIAEQKQKQGMEAHAGHPPGVPGGGDPADHAPKQTATRYKTLDSKGHYKSGTLDSRTYGRPDKEAPAPPPPPPPPELNRSERELNKMCGISASDIDKYSRIMFPVCFICFNLMYWIIYLHISDIVADDIVMLEPDK

***Apis mellifera* RDL-wild-type**

MSFHAASWSFALLAATVALLPATHRAPFAQAATGGGSMLNDVNISAILDSFSVSYDKRVRPNYGGPPVEVGVTMYVLSISSLSEVKMDFTLDFYFRQFWTDPRLAFKKRTGVETLSVGSEFIKNIWVPDTFFVNEKQSYFHIATTSNEFIRIHHSGSITRSIRLTITASCPMNLQYFPMDRQLCHIEIESFGYTMRDIRYKWNEGPNSVGVSNEVSLPQFKVLGHRQRAMEISLTTGNYSRLACEIQFVRSMGYYLIQIYIPSGLIVIISWVSFWLNRNATPARVALGVTTVLTMTTLMSSTNAALPKISYVKSIDVYLGTCFVMVFASLLEYATVGYMAKRIQMRKNRFQKIAESMKTARENPGPPGVPGDHGDHAPKQTEVRFKVHDPKAHSKGGTLENTINGRADEEAAPAPQHLIHPGKDINKLYGMTPSDIDKYSRIVFPVCFVCFNLMYWIIYLHISDVVADDLVLLEEAK

***Apis mellifera* RDL-G3’M$_{TMD3}$**

MSFHAASWSFALLAATVALLPATHRAPFAQAATGGGSMLNDVNISAILDSFSVSYDKRVRPNYGGPPVEVGVTMYVLSISSLSEVKMDFTLDFYFRQFWTDPRLAFKKRTGVETLSVGSEFIKNIWVPDTFFVNEKQSYFHIATTSNEFIRIHHSGSITRSIRLTITASCPMNLQYFPMDRQLCHIEIESFGYTMRDIRYKWNEGPNSVGVSNEVSLPQFKVLGHRQRAMEISLTTGNYSRLACEIQFVRSMGYYLIQIYIPSGLIVIISWVSFWLNRNATPARVALGVTTVLTMTTLMSSTNAALPKISYVKSIDVYL

MTCFVMVFASLLEYATVGYMAKRIQMRKNRFQKIAESMKTARENPGPPGVPGDHGDHAPKQ
TEVRFKVHDPKAHSKGGTLENTINGRADEEAAPAPQHLIHPGKDINKLYGMTPSDIDKYSRIVF
PVCFVCFNLMYWIIYLHISDVVADDLVLLEEAK

***Drosophila melanogaster* RDL-wild-type**

MSDSKMDKLARMAPLPRTPLLTIWLAINMALIAQETGNKRIHTVQAATGGGSMLGDVNISAIL
DSFSVSYDKRVRPNYGGPPVEVGVTMYVLSISSLSEVKMDFTLDFYFRQFWTDPRLAYRKRPG
VETLSVGSEFIKNIWVPDTFFVNEKQSYFHIATTSNEFIRVHHSGSITRSIRLTITASCPMNLQYFP
MDRQLCHIEIESFGYTMRDIRYKWNEGPNSVGVSSEVSLPQFKVLGHRQRAMEISLTTGNYSR
LACEIQFVRSMGYYLIQIYIPSGLIVVISWVSFWLNRNATPARVALGVTTVLTMTTLMSSTNAA
LPKISYVKSIDVYLGTCFVMVFASLLEYATVGYMAKRIQMRKQRSMAIQKIAEQKKQQLDGA
NQQQANPNPNANVGGPGGVGVGPGGPGGPGGGVNVGVGMGMGPEHGHGHGHHAHSHGHP
HAPKQTVSNRPIGFSNIQQNVGTRGCSIVGPLFQEVRFKVHDPKAHSKGGTLENTVNGGRGGP
QSHGPGPGQGGGPPGGGGVGGGGGGPPEGGGDPEAAVPAHLLHPGKVKKDINKLLGITPSDI
DKYSRIVFPVCFVCFNLMYWIIYLHVSDVVADDLVLLGEE

***Drosophila melanogaster* RDL-G3’M$_{TMD3}$**

MSDSKMDKLARMAPLPRTPLLTIWLAINMALIAQETGNKRIHTVQAATGGGSMLGDVNISAIL
DSFSVSYDKRVRPNYGGPPVEVGVTMYVLSISSLSEVKMDFTLDFYFRQFWTDPRLAYRKRPG
VETLSVGSEFIKNIWVPDTFFVNEKQSYFHIATTSNEFIRVHHSGSITRSIRLTITASCPMNLQYFP
MDRQLCHIEIESFGYTMRDIRYKWNEGPNSVGVSSEVSLPQFKVLGHRQRAMEISLTTGNYSR
LACEIQFVRSMGYYLIQIYIPSGLIVVISWVSFWLNRNATPARVALGVTTVLTMTTLMSSTNAA
LPKISYVKSIDVYLMTCFVMVFASLLEYATVGYMAKRIQMRKQRSMAIQKIAEQKKQQLDGA
NQQQANPNPNANVGGPGGVGVGPGGPGGPGGGVNVGVGMGMGPEHGHGHGHHAHSHGHP
HAPKQTVSNRPIGFSNIQQNVGTRGCSIVGPLFQEVRFKVHDPKAHSKGGTLENTVNGGRGGP
QSHGPGPGQGGGPPGGGGVGGGGGGPPEGGGDPEAAVPAHLLHPGKVKKDINKLLGITPSDI
DKYSRIVFPVCFVCFNLMYWIIYLHVSDVVADDLVLLGEE

***Tetranychus urticae* RDL-wild-type**

MMMLMLMLLLKPISLLCSLCCLPLLSLTMLIIPAFGHNWQSHGYQGDQPHEFGHTNGDIGSNI
SQILNSFFSSGYDKRVRPNYGGPPVKVGVTMHILTISSVSEVQMDFTADFYFRQLWRDSRLSFK
ARHGISQILVDAEVADKIWVPDTFFANEKQAYFHEATTKNTFLRISHDGQVLRSIRLTVTASCP
MNLQYFPMDRQKCNIEIESYGYSMTDIIYNWVDENAVKIDSNLMLPQFSIASIRQSWKYISLTT
GNYSRLMCEIQLTRSMGYYMIQIYVPASLIVIISWVSFWLHRNATPARVHLGVITVLTMTTLMS
STNSQLPKISYVKSIDVFLGTCFVMVFAALLEYAAVGYIGKRISMRKNRFQQLAKAAEEKRRK
LVEAAAAAAAAAAAATSCEPIHLSSSDPSGPQSHPINCNNSNNPSSINISTTTNPMNINNINSNG
TNNNNNNNNPNNNNNLNNINNSNNGSSITTLTTVATAAGGNVSSVGPMGSTGSTFNQTTGQL
VVDSALHGSGLTGTSVCSASATVGVPSVPKHQLYQQQQGQPHHHLTHHHRSSYQQHHQHLQ
QQLQQQQQQHQSIGTLTSVSNLGVGLSVPSNLVTYDFSGNGLSGGSGNLMMTTNPEMINCSL
GPGGHGGSMGSMPLGCSRDDQDQETLVTHVGHYATLRRPLLDRSSLPCKGAASSGVVGVTG
GTSVGSTGGSMPGQPNQKQFLPHRPQEVRLEMVGSKMTPVAQTSGSMDGVNHHQPGGLSSSS
IGRIPGHFTTNLHRFTAVPPKNLNNLFGVSPSDIDKYSRVVFPVCFVCFNLMYWIIFLHISSILEP
GADEES

Tetranychus urticae **RDL-G3'M$_{TMD3}$**

MMMLMLMLLLKPISLLCSLCCLPLLSLTMLIIPAFGHNWQSHGYQGDQPHEFGHTNGDIGSNI
SQILNSFFSSGYDKRVRPNYGGPPVKVGVTMHILTISSVSEVQMDFTADFYFRQLWRDSRLSFK
ARHGISQILVDAEVADKIWVPDTFFANEKQAYFHEATTKNTFLRISHDGQVLRSIRLTVTASCP
MNLQYFPMDRQKCNIEIESYGYSMTDIIYNWVDENAVKIDSNLMLPQFSIASIRQSWKYISLTT
GNYSRLMCEIQLTRSMGYYMIQIYVPASLIVIISWVSFWLHRNATPARVHLGVITVLTMTTLMS
STNSQLPKISYVKSIDVFLMTCFVMVFAALLEYAAVGYIGKRISMRKNRFQQLAKAAEEKRRK
LVEAAAAAAAAAAAATSCEPIHLSSSDPSGPQSHPINCNNSNNPSSINISTTTNPMNINNINSNG
TNNNNNNNNPNNNNNLNNINNSNNGSSITTLTTVATAAGGNVSSVGPMGSTGSTFNQTTGQL
VVDSALHGSGLTGTSVCSASATVGVPSVPKHQLYQQQQGQPHHHLTHHHRSSYQQHHQHLQ
QQLQQQQQQHQSIGTLTSVSNLGVGLSVPSNLVTYDFSGNGLSGGSGNLMMTTNPEMINCSL
GPGGHGGSMGSMPLGCSRDDQDQETLVTHVGHYATLRRPLLDRSSLPCKGAASSGVVGVTG
GTSVGSTGGSMPGQPNQKQFLPHRPQEVRLEMVGSKMTPVAQTSGSMDGVNHHQPGGLSSSS

IGRIPGHFTTNLHRFTAVPPKNLNNLFGVSPSDIDKYSRVVFPVCFVCFNLMYWIIFLHISSILEP
GADEES
